# Adhesion-driven rigidity transition decoupled from density-driven jamming triggers epithelial organization in embryonic tissues

**DOI:** 10.1038/s41567-026-03276-6

**Published:** 2026-06-02

**Authors:** Laura Rustarazo-Calvo, Cristina Pallares-Cartes, Adrián Aguirre-Tamaral, Elisa Floris, Maximilian Hingerl, Camilla Autorino, Arif Ul Maula Khan, Bernat Corominas-Murtra, Nicoletta I. Petridou

**Affiliations:** 1 European Molecular Biology Laboratory Heidelberg, Developmental Biology Unit, Heidelberg, Germany; 2 Collaboration for Joint PhD Degree between EMBL and Heidelberg University, Faculty of Biosciences, Heidelberg, Germany; 3 Department of Biology, University of Graz, Graz, Austria

**Keywords:** Biophysics, Soft materials, Statistical physics, Biological physics, Phase transitions and critical phenomena

## Abstract

The active regulation of tissue material properties via phase transitions is central in morphogenesis. Transitions occur abruptly at critical points in different control parameters, such as cell density, shape or adhesion. Whether these parameters are interdependent, and perform redundant or distinct functions, is unknown. Here we show that depending on the co-regulation of multiple control parameters, a tissue not only tunes its deformability but also its morphogenetic trajectory. We theoretically define a phase diagram capturing the material states of zebrafish pluripotent tissues undergoing epiboly—a tissue movement occurring during gastrulation—and show that they simultaneously cross critical points in cell density, connectivity and adhesion strength. We then combine optogenetics, biophysical measurements and quantitative morphometrics to independently modulate each parameter in vivo, and identify adhesion as the main determinant of tissue rheology. Further decoupling adhesion from density and inducing adhesion-driven rigidification in unjammed pluripotent tissues is sufficient to switch their morphogenetic program and trigger epithelial organization. This switch in tissue reorganization is achieved via tricellular junction formation, followed by lumenogenesis and the initiation of apical polarity. Our work reveals that the nonlinear dynamics of emergent tissue mechanics are mechanisms of tissue organization and morphogenesis.

## Abstract (web summary)

Tissue phase transitions are key to morphogenesis. Now adhesion-driven rigidification is identified as key to epithelial organization but only if uncoupled from density-driven jamming.

## Main

The active regulation of tissue material properties has been recently identified as a key factor establishing spatial and temporal deformation patterns during tissue spreading and folding, body axis elongation, wound healing and metastasis^1–9^. Recent work has shown that tissue material properties abruptly shift between discrete material regimes, for example, solid like versus fluid like, irrespective of force application^10–12^. The emergence of collective tissue phases—and the transitions therein—resemble phases of inert materials, where changes in the material state nonlinearly occur when a control parameter describing the interactions of the microscopic constituents reaches a critical point^13–15^. In contrast to inert materials, living materials display a variety of microscopic properties serving as control parameters of tissue phase transitions, including cell shape, density, connectivity and contact dynamics^16^. The multiplicity of parameters determining the tissue material state raises the question of which parameters are primarily relevant.

Multiple microscopic cellular mechanisms controlling the macroscopic material state have been reported, both theoretically and experimentally. For example, cell shape, tension fluctuations and motility^4,17,18^, and both cell density and connectivity^18,19^, were shown to trigger material phase transitions in confluent and non-confluent tissues, respectively. Thus, simple cell parameters allow to diagnose tissue material states, which are regulated by a handful of molecular players, such as actomyosin contractility and adhesion molecules^20^. The co-existence of multiple control parameters suggests that they may be interdependent but also that slight adaptations of their values, either individually or in combination, could trigger novel types of phase transition, possibly linked to biological function. This raises several questions. (1) Which control parameters are interdependent or redundant? (2) Can they be uncoupled? (3) Does each type of phase transition serve additional functions besides defining tissue deformability, depending on the control parameters used^21^?

We tackle these fundamental questions in the early zebrafish embryo, whose pluripotent blastoderm has been previously shown to undergo a rigidity phase transition, required for its spreading^5,19^. We theoretically derive a new critical point in contact surface tensions, which we empirically validate within the developing embryo, in combination with previously established critical points in cell density and connectivity^19,22,23^. We experimentally tune the multiple control parameters independently and disentangle their contributions to both tissue material state and organization. We conclude that contact surface tension is the major driver of tissue solidification and, when uncoupled from changes in density, it triggers epithelial organization in pluripotent tissues, both at a morphological and molecular level. We, therefore, show that material phase transitions impact the morphogenetic potential of pluripotent embryonic tissues.

## Multiple control parameters are coupled during an in vivo material phase transition

**Fig. 1:**
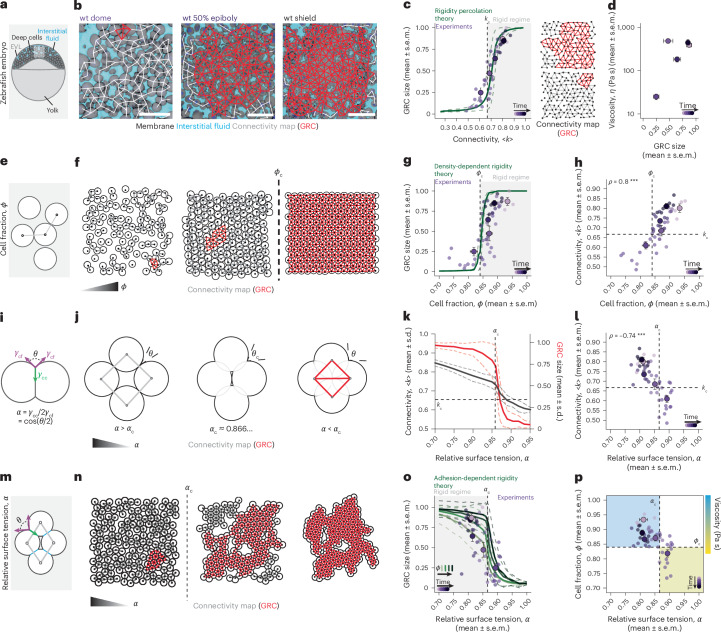
Critical points in cell fraction, contact surface tensions and cell connectivity are crossed simultaneously during a rigidity transition in the zebrafish blastoderm. **a**, Schematic of the zebrafish dome-stage embryo. **b**, Example 2D confocal sections of the second deep-cell layer of the blastoderm, overlaid with their connectivity and rigidity maps at dome, 50% epiboly and shield stages. Interstitial fluid is marked with dextran-647, nuclei with H2A-BFP, H2A-mCherry or H2B-GFP and membranes with α-catenin-citrine or Lyn-Tomato. **c**, Plot of the fraction of the network occupied by GRC as a function of normalized connectivity <$k$> in the simulated random 2D triangular lattices like the example networks shown in right panel, with the overlaid experimental data from high until shield-stage embryos quantified for the fraction of the network occupied by GRC (embryos per stage: high *n* = 4, sphere *n* = 11, dome *n* = 14, 50% epiboly *n* = 11, shield *n* = 6). The grey-shaded area indicates the network rigid regime above the critical connectivity point (dashed line). **d**, Plot of the central blastoderm viscosity values in zebrafish embryos from high to shield stage, as measured via MPA^5^, and as a function of GRC size (viscosity measurements, embryos per stage: high *n* = 30, sphere *n* = 26, dome *n* = 30, 50% epiboly *n* = 15, shield *n* = 16; GRC measurements as in **c**). **e**, Schematic of the density-dependent connectivity. **f**, Numerical simulations and overlaid connectivity and rigidity maps of jammed hard discs performed using the Lubachevsky–Stillinger algorithm^37^ at different fraction $\phi$ values. Rigidity emerges at ${\phi }_{c}\approx 0.84$. **g**, Plot of the fraction of the network occupied by the GRC as a function of cell fraction $\phi$ for the simulations shown in **f**, with overlaid experimental data from zebrafish embryos from high until shield stage (same as in **c**). **h**, Plot of normalized connectivity <$k$> as a function of cell fraction $\phi$ from the experimental data shown in **c**. **i**, Relative surface tension $\alpha$ is defined by the Young–Dupré relation and can be calculated from the angle $\theta$ formed at the contact edge. **j**, Numerical simulations for the morphology of a four-disc rhombus cluster at different relative surface tension $\alpha$ values. By decreasing $\alpha$ below ${\alpha }_{c}$, a new contact is formed, rigidifying the small cluster. **k**, Plot of normalized connectivity <$k$> (left *y* axis, grey) and fraction of the network occupied by GRC (right *y* axis, red) as a function of the relative surface tension $\alpha$ for simulated cell arrays like those depicted in **n**, showing a sudden increase in connectivity and GRC size at ${\alpha }_{c}$. **l**, Plot of normalized connectivity <$k$> as a function of relative surface tension $\alpha$ from the experimental data shown in **c**. **m**, Schematic diagram of adhesion-dependent connectivity. **n**, Numerical simulations and overlaid connectivity and rigidity maps of cell arrays with $\phi < {\phi }_{c}$ at different relative surface tension $\alpha$ values. Rigidity emerges at ${\alpha }_{c}\approx 0.866.$ **o**, Plot of the fraction of the network occupied by the GRC as a function of relative surface tension $\alpha$ for the simulations shown in **n**, performed for different cell fraction $\phi$ values below ${\phi }_{c}$, and overlaid experimental data from zebrafish embryos from high until shield stage (same as in **c**). **p**, Phase diagram of tissue viscosity with coordinates cell fraction $\phi$ and relative surface tension $\alpha$ and overlaid experimental data from **h** and **l** showing that wt embryos are constrained to two out of the four regimes. Cyan-shaded areas indicate high viscosity and yellow-shaded areas indicate low viscosity. Floppy areas are illustrated in grey and GRC, in red. Light purple colour, early stage; dark purple colour, late stage; dashed lines, critical points. Statistics: *ρ*, Spearman correlation; *P* < 0.0001 (**h**); *P* < 0.0001 (**l**). Scale bars, 50 µm (**b**). Source data

**Extended Data Fig. 1:**
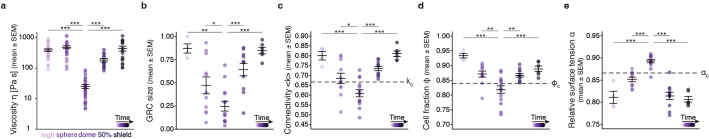
Quantitative analysis and theoretical relationships between multiple control parameters and tissue rigidity and viscosity. **(a-e)** Plots of experimental data from high- until shield-stage wildtype embryos quantified for (a) blastoderm viscosity as measured via micropipette aspiration, (b) the fraction of the network occupied by the GRC, (c) normalized connectivity <$k$>, (d) cell fraction $\phi$, and (e) relative surface tension $\alpha$. Embryo numbers as in Fig. 1. Dashed lines: critical points. Statistics: Kruskal-Wallis with Dunn’s post-hoc test (Bonferroni correction) (a) global *p* < 0.0001, all individual comparisons *p* < 0.0001, (b) global < 0.0001, individual comparisons: high *p* ≈ 0.0029, sphere *p* ≈ 0.0375, epiboly *p* ≈ 0.0003, germ ring *p* ≈ 0.0005; ANOVA with Dunnett’s post-hoc test (c) global *p* < 0.0001, individual comparisons: high *p* < 0.0001, sphere *p* ≈ 0.0185, epiboly *p* < 0.0001, germ ring *p* < 0.0001, (d) global *p* < 0.0001, individual comparisons: high *p* < 0.0001, sphere *p* ≈ 0.0017, epiboly *p* ≈ 0.0041, germ ring *p* ≈ 0.0005, (e) global *p* < 0.0001, all individual comparisons *p* < 0.0001. Source data

To gain an insight into which cell parameters regulate tissue material phase transitions in vivo, we quantified several cellular properties during zebrafish embryogenesis. The early embryo consists of a blastoderm positioned atop a yolk cell (Fig. 1a). The blastoderm comprises an epithelial monolayer (enveloping layer (EVL)) enclosing a multilayered, semiconfluent deep-cell mass immersed in interstitial fluid (Fig. 1a,b). We have previously measured the viscosity of the central deep-cell mass using micropipette aspiration (MPA) and showed that at pluripotent stages (high, sphere), it has high viscosity, at the onset of morphogenesis (dome), viscosity abruptly decreases and, by gastrulation (50% epiboly, shield stage), it increases again^5,24^ (Extended Data Fig. 1a). Using the framework of rigidity percolation theory, we identified that tissue fluidization can be interpreted as a rigid-to-floppy phase transition occurring at a critical point in cell connectivity^19^. This framework links the material state of a network to the size of the giant rigid cluster (GRC), which is the largest subgraph in a network made of viscoelastic links that cannot be deformed without an energy cost (Fig. 1c, left and right)^2,25–27^. A network is generically rigid when almost all of it satisfies the rigidity conditions of the Geiringer–Laman Theorem^26,28,29^ (Box 1). When connectivity <$k$> reaches the critical value of Maxwellian rigidity <${k}_{{\rm{c}}}$>, namely, the isostatic point, the GRC abruptly increases from a vanishing size at the subcritical phase, to spanning almost all the network at the supercritical phase (Fig. 1c, left and right). Abstracting three-dimensional (3D) embryonic tissues as two-dimensional (2D) cellular networks is sufficient to map their material state to GRC size^19^ (Fig. 1b–d). The pluripotent embryo has connectivity above the critical point and a big GRC; at the dome stage, it crosses the critical point, the GRC almost vanishes and the tissue fluidizes, and then it rigidifies again, reflecting the tissue viscosity measurements (Fig. 1b–d and Extended Data Fig. 1b,c)^19^. Previous theoretical work in hard and soft spheres showed that connectivity changes as a function of both packing fraction and the attractive/repulsive nature of the interactions, triggering jamming transitions^18,23,30^. Assuming that the biological analogues are cell density and cell–cell adhesion strength, we explore how density-dependent versus adhesion-dependent phase transitions contribute to changes in connectivity and tissue material state.

The standard theory of jamming transitions in 2D frictionless hard discs and soft spheres predicts that beyond a critical point in cell fraction ${\phi }_{c}$ (${\phi }_{c}\approx 0.84$)^31,32^, the rheological response of the tissue is determined by the fact that cells are jammed, stresses propagate throughout large tissue fractions and any deformation implies an energy penalty^25,30,31,33^. Concerning the topology of cell–cell contacts, the isostatic point in jamming ${\phi }_{c}$ coincides with the emergence of generic rigidity (Fig. 1e,f)^34^. At ${\phi }_{c}\,$, the cell–cell contact network crosses the critical point <${k}_{{\rm{c}}}$> (<${k}_{c}$> $\approx$ 4 in two dimensions), leading to rigid cluster percolation (Fig. 1f,g)^22,26^. When quantifying the cell fraction, connectivity, GRC size^35,36^ and tissue viscosity in zebrafish, we observe that wild-type (wt) tissues with cell fraction above the critical point display connectivity above the rigidity percolation threshold, a large GRC and high viscosity (Fig. 1g,h and Extended Data Fig. 1b–d). These results suggest that the rigidification of embryonic tissues follows the physics of the density-dependent jamming of granular materials.

Previous reports showed that jamming-dependent rigid cluster percolation appears both when cell–cell contacts are passive (as in hard, non-attractive or repulsive spheres^37,38^) or active (as in cell–cell contact networks with active adhesion mechanisms^19,23,30^). To explore the contribution of active adhesion to rigidity percolation, we theoretically approached cell–cell adhesive interactions within the theoretical framework provided by the physics of foams^39^. We used the non-dimensional parameter $\alpha$, defined as the ratio between cell–cell ${\gamma }_{{\rm{cc}}}$ and cell–fluid ${\gamma }_{{\rm{cf}}}$ surface tensions, which can be estimated from the angle $\theta$ formed at the contact edge between two cells^19,39–43^ (Fig. 1i and Supplementary Fig. 1). The connection between surface tensions and geometry is given by the Young–Dupré law, which, for equally sized cells under force-balance conditions, reads

1 $$\alpha =\,\frac{{\gamma }_{{\rm{cc}}}}{2{\gamma }_{{\rm{cf}}}}=\cos \left(\frac{\theta }{2}\right).$$

From this non-dimensional adhesion parameter, one can define a Hamiltonian that will drive the relaxation to equilibrium of the system under a relative surface tension $\alpha$:

2 $$H\left(\alpha \right)=\alpha \mathop{\sum}\limits_{ij}{w}_{{\rm{ij}}}+\frac{1}{2}\mathop{\sum}\limits_{i}{a}_{i}+{\rm{K}}\mathop{\sum }\limits_{i}{({A}_{i}-{A}_{0})}^{2},$$

where ${w}_{{\rm{ij}}}$ is the contact length between cells *i* and $j$, ${a}_{i}$ is the length of cell *i* exposed to the interstitial fluid, ${A}_{i}$ is the area of cell *i*, ${A}_{0}$ is the area of cells in force balance and *K* is the constant accounting for the penalization of area deviations. We assume that all cells have the same preferred area ${A}_{0}$ and that $K$ is big enough, such that area conservation can be considered as a boundary condition of the energy minimization problem. To examine how rigid cluster percolation is influenced by $\alpha$, we mathematically explored the behaviour of a four-cell floppy motif under different $\alpha$ values (Fig. 1j). Using the Young–Dupré law, we analytically identified a critical point ${\alpha }_{c}$, at which a new contact is formed and the four-cell motif rigidifies (Fig. 1j, Box 1 and Supplementary Fig. 2). The explicit numerical value of ${\alpha }_{c}$ can be approximated to ${\alpha }_{{\rm{c}}}\approx 0.866$.

To address if ${\alpha }_{c}$ can explain rigidification in an arbitrary array of cells, we generated in silico random tilings^44^ (Lubachevsky–Stillinger Algorithm–Random Disk Spreading (LSA-RDS) is described in the Supplementary Information) at the hard-disc regime ($\alpha =1$, no adhesion) with cell fraction below the critical jamming fraction ($\phi < {\phi }_{c}$), and for which we verified that the GRC size is negligible (Fig. 1m,n). Then, we decreased $\alpha$ in a quasi-static way using the Surface Evolver software^45^ following the Hamiltonian provided by equation (2). Once the system reached the optimal configuration, we computed the GRC size. At ${\alpha }_{c}$, a jump in the average connectivity above <${k}_{c}$> and the corresponding transition in GRC size are observed (Fig. 1k,n,o). In consequence, the relation between surface tensions *α* emerges as a control parameter, driving a rigidity transition with a critical point at ${\alpha }_{c}\approx 0.866$. Importantly, when quantifying contact surface tensions in vivo, we observe that tissues of high viscosity, large GRC, and with connectivity and cell fraction above their corresponding critical points also have $\alpha$ below ${\alpha }_{c}\,$(Fig. 1l,o and Extended Data Fig. 1e).

On the basis of these findings, we propose a 2D $(a,\,\phi )$ phase diagram of the tissue material state organized around the critical points in relative surface tension ${\alpha }_{c}$ and cell fraction ${\phi }_{c}$ (Fig. 1p). The wt embryos are constrained to two out of the four potential material regimes: the top-left regime, corresponding to topologically rigid tissues of high viscosity, and the bottom-right regime, corresponding to topologically floppy tissues of low viscosity (Fig. 1p). During this transition, embryos simultaneously cross the critical points ${\phi }_{c}$, ${\alpha }_{c}$ and <${k}_{c}$> by coupling changes in cell fraction, contact surface tensions and cell connectivity. This raises the questions of whether these control parameters can be uncoupled, and how each parameter contributes to the tissue rheological state.

Box 1 Rigidity foundations Topological conditions/Geiringer–Laman theorem

A network ${\mathrm{G}} \acute{} ({\mathrm{V}} \acute{} ,{\mathrm{E}} \acute{}\,)$ embedded in a 2D space will be generically rigid if there is a connected, spanning subgraph $G(V,E)$, with $E\subseteq {\rm{E}} \acute{}$, by which
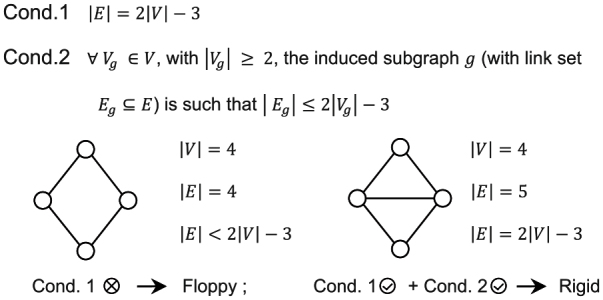

Identification of the critical point triggering rigidity
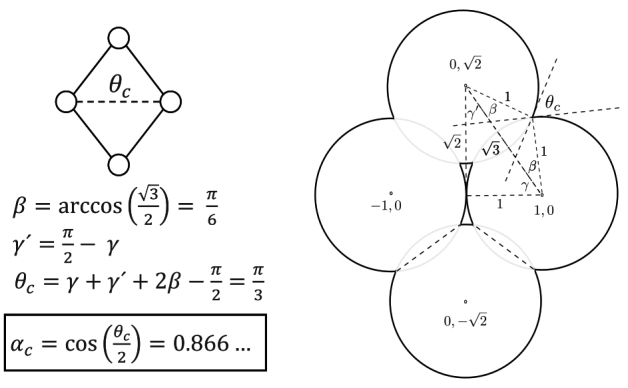

## Relative surface tension is the major control parameter of the tissue material state in vivo

**Fig. 2:**
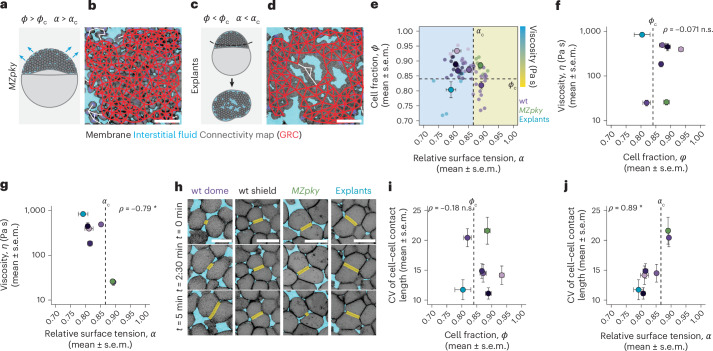
Decoupling the contributions of cell fraction and contact surface tensions to the tissue material state. **a**,**b**, Schematic (**a**) and example 2D confocal section (**b**) at the second deep-cell layer of the blastoderm of *MZpky* embryos, in which the interstitial fluid leaks out. Membranes labelled with membrane-GFP, nuclei with H2A-BFP and interstitial fluid with dextran-647. **c**,**d**, Schematic (**c**) and example 2D confocal section (**d**) of the deep-cell tissue of blastoderm explants. Membranes labelled with α-catenin-citrine, nuclei with H2A-mCherry and interstitial fluid with dextran-647. **e**, Phase diagram of tissue viscosity with coordinates cell fraction $\phi$ and relative surface tension $\alpha$, with experimental data from wt, *MZpky* and explanted blastoderms (n = 6 *MZpky* embryos, *n* = 6 explants; wt as that shown in Fig. 1c). Cyan-shaded areas indicate high viscosity and yellow-shaded areas indicate low viscosity. **f**,**g**, Plots of blastoderm viscosity values for the conditions shown in **e**, as a function of cell fraction $\phi$ (**f**) and relative surface tension $\alpha$ (**g**) (viscosity measurements: wt as shown in Fig. 1d; n = 16 *MZpky* and *n* = 6 explants; cell fraction $\phi$ and relative surface tension $\alpha$ as in **e**). **h**, Representative high-resolution 2D confocal sections from time-lapse imaging over 10 min at 10-s intervals (images shown at *t* = 0, 150 and 300 s) of wt dome and shield stage, *MZpky* and explants. The yellow-shaded line marks the cell–cell contact length. Membranes labelled with α-catenin-citrine or membrane-GFP and interstitial fluid with dextran-647. **i**,**j**, Plots of the CV in cell–cell contact length for the conditions shown in **h** as a function of cell fraction $\phi$ (**i**) and relative surface tension $\alpha$ (**j**) (embryos *N* and contacts *n* per stage and condition: wt high *N* = 3, *n* = 13; wt sphere *N* = 3, *n* = 12; wt dome *N* = 8, *n* = 39; wt 50% epiboly *N* = 7, *n* = 19; wt germ ring to shield *N* = 8, *n* = 36; *MZpky* *N* = 6, *n* = 27; explants *N* = 5, *n* = 21). Dashed lines, critical points. Statistics: *P* ≈ 0.91 (**f**), *P* ≈ 0.048 (**g**), *P* ≈ 0.71 (**i**), *P* ≈ 0.012 (**j**). Scale bars, 50 µm (**b** and **d**); 20 µm (**h**). Source data

**Extended Data Fig. 2:**
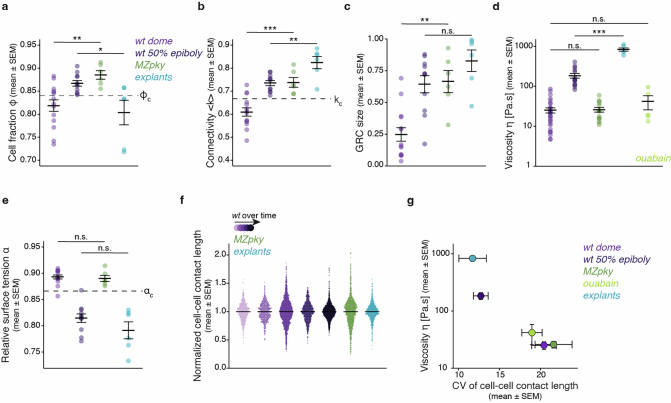
Quantitative analysis of cell and tissue biophysical properties during the rigidity transition. **(a-f)** Plots of (a) cell fraction $\phi$, (b) normalized connectivity <$k$>, (c) fraction of the network occupied by the GRC, (d) blastoderm viscosity, (e) relative surface tension $\alpha$, and (f) cell-cell contact lengths for *wt* dome-stage, *wt* 50% epiboly-stage, *MZpky*, explants and ouabain dome-stage embryos (only in (d)). **(g)** Plot of blastoderm viscosity as a function of the coefficient of variation in cell-cell contact length. Embryo and contact numbers for *wt*, explants and *MZpky* as in Figs. 1 and 2. Embryo and contact numbers for ouabain embryos as in Fig. 3, for viscosity *n* = 5 embryos. Dashed lines: critical points. Statistics: Two-sided Student’s t-test for *wt* dome vs. *MZpky* (a) *p* ≈ 0.0042, (b) *p* ≈ 0.0006, (e) *p* ≈ 0.6940 and *wt* epiboly vs. explant (b) *p* ≈ 0.0023, (c) *p* ≈ 0.1164, (e) *p* ≈ 0.1726; two-sided Mann-Whitney U-test for *wt* dome vs. *MZpky* (c) *p* ≈ 0.0044 and *wt* epiboly vs. explant (a) *p* ≈ 0.0138, (d) *p* ≈ 0.0005; Kruskal-Wallis with Dunn’s post-hoc test (Bonferroni correction) for comparisons to dome (d) global *p* ≈ 0.4544. Source data

To address the above questions, we experimentally uncoupled the cell fraction, relative surface tension and connectivity, and measured the tissue viscosity using MPA^5,24^. To achieve this, we modulated the interstitial fluid amount by interfering with the EVL without affecting the adhesive properties of the deep-cell tissue using two approaches: (1) mutant *MZpky* embryos^46^ in which the EVL is disrupted and the interstitial fluid leaks out, leading to tissues of high cell fraction (Fig. 2a,b, Extended Data Fig. 2a and Extended Data Table 1); and (2) embryonic explants^47,48^ in which the blastoderm is excised and, while the EVL heals to enclose the deep-cells, more fluid is accumulated, lowering the cell fraction (Fig. 2c,d, Extended Data Fig. 2a and Extended Data Table 1). Both manipulations decouple cell fraction and contact surface tensions, with *MZpky* embryos having cell fraction above ${\phi }_{c}$, connectivity above <${k}_{c}$> and relative surface tension above ${\alpha }_{c},$ falling in the top-right regime of the phase diagram, and zebrafish explants having cell fraction below ${\phi }_{c}$, connectivity above <${k}_{c}$> and relative surface tension below ${\alpha }_{c},$ falling in the bottom-left regime (Fig. 2e and Extended Data Fig. 2a,b,e). Remarkably, MPA experiments revealed that *MZpky* embryos display low viscosity despite appearing topologically rigid and, conversely, zebrafish explants are topologically rigid and highly viscous despite having cell fraction below ${\phi }_{c}$ (Fig. 2f,g and Extended Data Fig. 2c,d). These results show that when cell density and adhesion changes are uncoupled, the tissue material state is determined by the cell–cell adhesion strength.

To understand why $\alpha$ is a better predictor of the tissue material state than cell fraction, we examined the relationship between $\alpha$ and active cell parameters, such as cell–cell contact length fluctuations (CLFs)^18^ (Fig. 2h and Extended Data Fig. 2f). We observe a dependency between relative surface tension, CLFs and tissue viscosity, where all tissues above ${\alpha }_{c}$ display large CLFs and are fluidized, irrespective of their cell fraction (Fig. 2i,j and Extended Data Fig. 2g). This suggests that even when $\phi > {\phi }_{c}$ and <$k$> > <${k}_{c}$>, cell–cell contacts are highly dynamic if $\alpha > {\alpha }_{c}$, allowing the tissue to deform, explaining why $\alpha$ is an accurate proxy for the tissue material response. These results show that although early embryonic tissues couple density-dependent and adhesion-dependent material phase transitions, their material state is only determined by the latter, raising the question of the function of control parameter (un)coupling in morphogenesis.

## Precise engineering of tissue material states via uncoupling the multiple control parameters

**Extended Data Fig. 3:**
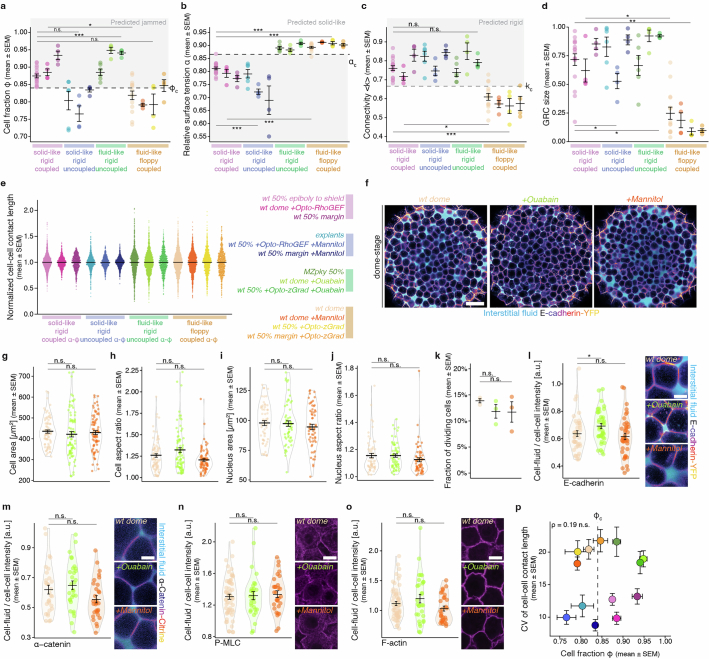
Quantification of control and order parameters in all experimental conditions and characterization of the osmoregulation tools. **(a-e)** Plots of quantifications of cell fraction $\phi$ (a), relative surface tension $\alpha$ (b), normalized connectivity <$k$> (c), fraction of the network occupied by the GRC (d), and normalized cell-cell contact lengths (e) for all experimental conditions listed (embryo and contact numbers as in Fig. 3). (**f**) Exemplary confocal sections of dome-stage embryos endogenously tagged for E-cadherin-YFP and with labeled interstitial fluid, for *wt*, ouabain- and mannitol-treated conditions. (**g-k**) Plots of quantifications of cell area (g), cell aspect ratio (h), nucleus area (i), nucleus aspect ratio (j), and mitotic fraction (k) for the experimental conditions shown in (f) (g, h: *N* = 4 embryos and *n* = 80 cells per condition; i, j: *N* = 3 embryos and *n* = 60 nuclei per condition; k: *N* = 3 embryos per condition, with *n* = 606 cells for *wt*, *n* = 611 cells for Ouabain, and *n* = 534 cells for Mannitol). (**l, m**) (left) Plots of quantifications of E-cadherin (l) and α-catenin (m) distribution at the cell-fluid vs cell-cell interfaces, and (right) exemplary high-magnification images for the experimental conditions *wt*, ouabain- and mannitol-treated dome-stage zebrafish embryos from knock-in lines for E-cadherin-YFP (l) and α-catenin-citrine (m) (l: *N* = 4 embryos and *n* = 40 cells per condition; m: *N* = 3 embryos and *n* = 30 cells per condition). (**n, o**) (left) Plots of quantifications of P-MLC (n) and F-actin (o) cell surface distribution, and (right) exemplary high-magnification images for the experimental conditions *wt*, ouabain- and mannitol-treated dome-stage zebrafish embryos immunostained for P-MLC (n) and F-actin (o) (for each staining, *wt* *N* = 6 embryos and *n* = 60 cells, ouabain *N* = 3 embryos and *n* = 30 cells, mannitol *N* = 3 embryos and *n* = 30 cells). (**p**) Plot of the coefficient of variation (CV) in cell-cell contact length for the conditions shown in (e) as a function of cell fraction $\phi$ (embryo and contact numbers as in Fig. 3). Statistics: Welch’s ANOVA with Games-Howell post-hoc test (a) global *p* < 0.0001, individual comparisons: *wt* dome *vs. wt* dome + Opto-RhoGEF *p* ≈ 0.0120, *wt* 50% epiboly to shield *vs. wt* 50% + Opto-RhoGEF + Mannitol *p* ≈ 0.0680, *wt* 50% epiboly to shield *vs. wt* 50% + Opto-zGrad *p* ≈ 0.285, *wt* 50% epiboly to shield *vs. wt* 50% + Opto-zGrad + Ouabain *p* < 0.0001; ANOVA with Tukey HSD post-hoc test (b) global *p* < 0.0001, all individual comparisons *p* < 0.0001, (c) global *p* < 0.0001, individual comparisons: *wt* dome *vs. wt* dome + Opto-RhoGEF *p* ≈ 0.0222, *wt* 50% epiboly to shield *vs. wt* 50% + Opto-RhoGEF + Mannitol *p* ≈ 0.9971, *wt* 50% epiboly to shield *vs. wt* 50% + Opto-zGrad *p* < 0.0001, *wt* 50% epiboly to shield *vs. wt* 50% + Opto-zGrad + Ouabain *p* ≈ 0.9456; Kruskal-Wallis with Dunn’s post-hoc test (Bonferroni adjustment) (d) global *p* < 0.0001, individual comparisons: *wt* dome *vs. wt* dome + Opto-RhoGEF *p* ≈ 0.0195, *wt* 50% epiboly to shield *vs. wt* 50% + Opto-RhoGEF + Mannitol *p* ≈ 0.0488, *wt* 50% epiboly to shield *vs. wt* 50% + Opto-zGrad *p* ≈ 0.0031, *wt* 50% epiboly to shield *vs. wt* 50% + Opto-zGrad + Ouabain *p* ≈ 0.0122, (g) global *p* ≈ 0.3357, (h) global *p* ≈ 0.0116, (j) global *p* ≈ 0.0637, (l) global *p* ≈ 0.0165, individual comparisons: Ouabain *p* ≈ 0.0232, Mannitol *p* ≈ 0.6101, (n) global *p* ≈ 0.7897, (o) global *p* ≈ 0.1789; ANOVA with Dunnett’s post-hoc test (i) global *p* ≈ 0.5311, (k) global *p* ≈ 0.5096, (m) global *p* ≈ 0.0572. Scale bars: 50 µm (f), 10 µm (l-o).; ρ: Spearman correlation (p) *p* ≈ 0.53. Source data

To explore if and how the control parameter coupling impacts the tissue morphogenetic outcome, we engineered the material state of wt embryos by either coupling or uncoupling the changes in cell fraction and relative surface tension. To do this, we combined methodologies that fine-tune the two control parameters in relation to their critical points in (1) wt dome-stage embryos, which have high relative surface tension ($\alpha > {\alpha }_{c}$), low cell fraction ($\phi < {\phi }_{c}$) and are topologically floppy (small GRC, <$k$> $< \,$<${k}_{c}$>) and rheologically fluid-like (large CLFs), and (2) wt 50% epiboly-stage embryos, which have low relative surface tension ($\alpha < {\alpha }_{c}$), high cell fraction ($\phi > {\phi }_{c}$) and are topologically rigid (big GRC, <$k$> > <${k}_{c}$>) and rheologically solid-like (small CLFs) (Extended Data Fig. 3a–e).

**Fig. 3:**
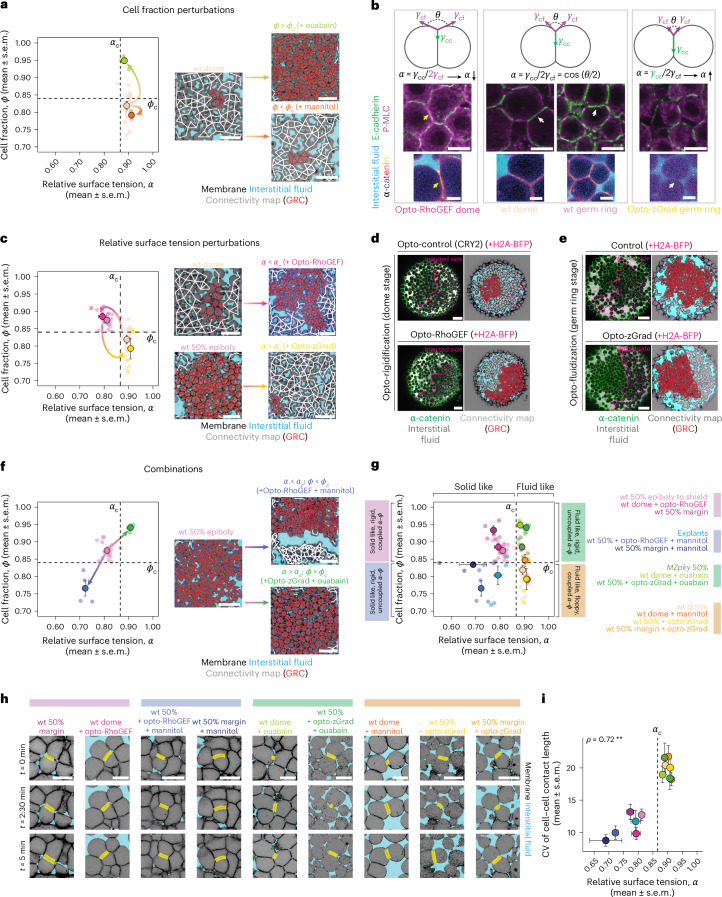
Precise engineering of tissue material states via independent fine-tuning of the control parameters. The data shown here are related to Supplementary Videos 1–3. **a**, Phase diagram of the cell fraction perturbation experiments, with coordinates cell fraction $\phi$ and relative surface tension $\alpha$ (left), and representative 2D confocal sections of the blastoderm with overlaid connectivity and rigidity maps (right) from dome-stage embryos, either untreated (wt) or osmotically altered with ouabain or mannitol to increase or decrease cell fraction $\phi$ in relation to ${\phi }_{c}$ (embryo numbers as in **g**). **b**, Schematic (top row) depicting how contact surface tensions are expected to change in the different experimental conditions and representative high-resolution confocal images of opto-RhoGEF dome-stage embryos (left), wt dome and wt germ ring stages (middle), and opto-zGrad germ ring-stage embryos (right) stained for E-cadherin and P-MLC (middle) or live imaged and labelled for α-catenin and interstitial fluid (bottom). The yellow and white arrowheads indicate P-MLC or α-catenin enrichment or absence at the cell–cell interface, respectively. **c**, Phase diagram with the parameters shown in **a** (left) and the representative 2D confocal sections of the blastoderm with overlaid connectivity and rigidity maps (right) from wt or optogenetically rigidified (opto-RhoGEF) dome-stage embryos and from wt or optogenetically fluidized (opto-zGrad) 50% epiboly-stage embryos (embryo numbers as in **g**). **d**, Example confocal sections of dome-stage embryos, in which one-half of the embryo was injected with H2A-BFP and CIBN-CAAX together with either CRY2 (opto-control) (top) or opto-RhoGEF (bottom), as well as their corresponding connectivity and rigidity maps (right). The magenta dashed line depicts the border between the injected and non-injected side, showing successful half-embryo rigidification only in the injected side of the opto-RhoGEF condition. **e**, Example confocal sections of germ ring-stage embryos, which were injected in half of the embryo with H2A-BFP alone (top) or together with opto-zGrad (bottom), together with their corresponding connectivity and rigidity maps (right). The magenta dashed line depicts the border between the injected and non-injected side, showing successful half-embryo fluidization only in the injected side of the opto-zGrad condition. **f**, Phase diagram with the parameters shown in **a** (left) and the representative 2D confocal sections of the blastoderm with overlaid connectivity and rigidity maps (right) from 50% epiboly-stage embryos, either wt or optogenetically rigidified in a hypertonic solution (opto-RhoGEF + mannitol) or optogenetically fluidized in a hypotonic solution (opto-zGrad + ouabain) (embryo numbers as in **g**). **g**, Phase diagram with the parameters shown in **a** with overlaid experimental data from different combinations of genetic, optogenetic and osmotic manipulations occupying the four material regimes of the phase diagram (embryo numbers: wt 50% epiboly to shield *n* = 17, explants *n* = 6, *MZpky* *n* = 6, wt dome *n* = 14, and *n* = 4 embryos for wt 50% + opto-zGrad, wt dome + opto-RhoGEF, wt 50% margin, wt 50% + opto-RhoGEF + mannitol, wt 50% margin + mannitol, wt dome + ouabain, wt 50% + opto-zGrad + ouabain, wt dome + mannitol, and wt 50% margin + opto-zGrad). **h**, Representative high-resolution 2D confocal sections from time-lapse imaging over 10 min at 10-s intervals (images shown at *t* = 0, 150 and 300 s) of the experimental conditions shown in **g**. The yellow-shaded line marks the cell–cell contact length. **i**, Plot of CV in cell–cell contact length for the conditions shown in **h** as a function of relative surface tension $\alpha$ (embryo numbers for $\alpha$ as in **g**; embryos *N* and contacts *n* for CLFs per stage and condition: wt 50% epiboly to shield *N* = 13, *n* = 45; wt dome + opto-RhoGEF *N* = 4, *n* = 18; wt 50% margin *N* = 4, *n* = 15; explants *N* = 5, *n* = 21; wt 50% + opto-RhoGEF + mannitol *N* = 3, *n* = 15; *wt* 50% margin + mannitol *N* = 3, *n* = 20; *MZpky* *N* = 6, *n* = 27; wt dome + ouabain *N* = 5, *n* = 35; wt 50% + opto-zGrad + ouabain *N* = 3, *n* = 15; wt dome *N* = 8, *n* = 39; wt dome + mannitol *N* = 6, *n* = 30; wt 50% + opto-zGrad *N* = 5, *n* = 20; wt 50% margin + opto-zGrad *N* = 3, *n* = 15). Dashed lines, critical points. Statistics: *P* ≈ 0.0075 (**i**). Membranes labelled with α-catenin-citrine or Lyn-Tomato, nuclei with H2A-BFP, H2B-GFP or H2A-mCherry and interstitial fluid with dextran-647. Scale bars, 50 µm (**a**, **c**, **d, e** and **f**); 10 µm (**b**); 20 µm (**h**). Source data

To increase the cell fraction, we induced a hypotonic environment by using ouabain, a Na^+^/K^+^-ATPase inhibitor, whereas to decrease cell fraction, we induced a hypertonic environment by injecting the sugar alcohol mannitol in the interstitial fluid^49,50^ (Extended Data Table 1). This approach has no off-target effects in the cell cytoskeleton or physiology (Extended Data Fig. 3f–o) and successfully reduces or increases interstitial fluid, which results in dome-stage embryos slightly changing their cell fraction just above or below ${\phi }_{c}$, respectively (Fig. 3a and Extended Data Fig. 3a).

To change the cell–cell adhesion strength, we established tools acting on relative surface tension (Extended Data Table 1). Previous studies showed that cell–cell contact formation is accompanied by an asymmetric localization of the adherens junction (AJ) components and actomyosin along the cell surface^40,51,52^. As cell–cell contacts mature, actomyosin is depleted from the cell–cell interface and contractility promotes contact stabilization^53,54^. Such differential localization is also observed in vivo^5,19,41,55,56^, with E-cadherin and α-catenin localizing at the cell–cell interface and F-actin and phosphorylated myosin light chain (P-MLC) at the cell–fluid interface (Fig. 3b and Extended Data Fig. 3l–o). Given that the cell–cell adhesion strength can be molecularly regulated by the interplay of AJs and actomyosin^5,40,41^, we sought to fine-tune the relative surface tension using optogenetics to either (1) enhance the actomyosin contractility and, thus, increase ${\gamma }_{{\rm{cf}}}$ and decrease $\alpha$ (Fig. 3b, left); or (2) deplete the AJ components and, consequently, increase ${\gamma }_{{\rm{cc}}}$ and $\alpha$ (Fig. 3b, right).

**Extended Data Fig. 4:**
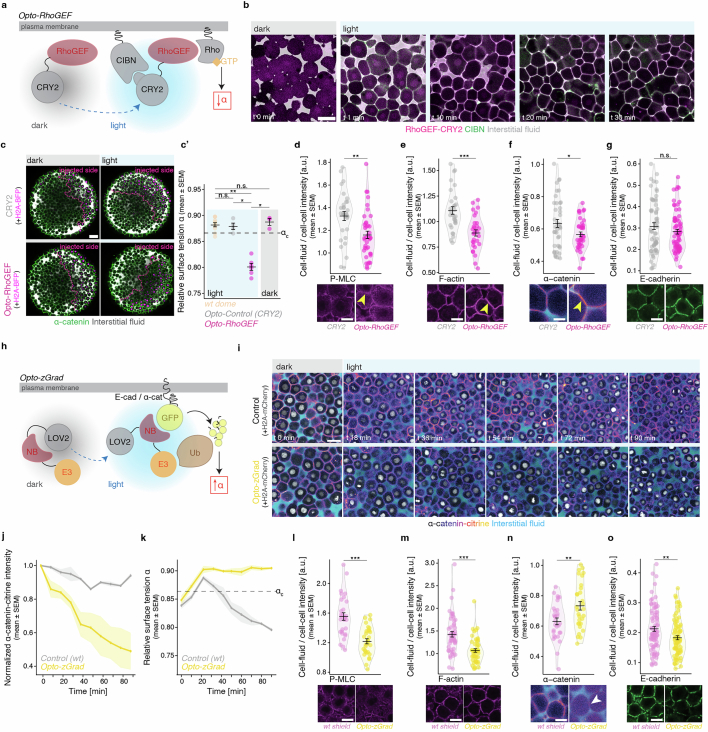
Quantification of the specificity and molecular effects of optogenetic tools. **(a)** Schematic diagram of the Opto-RhoGEF tool, which combines a membrane-tagged CIBN and RhoGEF-CRY2^57^. **(b)** Exemplary timelapse stills of Opto-RhoGEF illumination, showing a quick change in its subcellular localization from cytosolic (dark) to membrane (light), due to the binding of CRY2 to membrane-tagged CIBN. Interstitial fluid labeled with dextran-647. (**c**) Representative confocal sections of dome-stage embryos, which were co-injected in half of the embryo with H2A-BFP and Opto-Control (CRY2 alone) (top panel), or Opto-RhoGEF (bottom panel), in dark (left) and light (right) conditions. The magenta dashed line depicts the border between the injected and non-injected side. (**c’**) Quantification of the relative surface tension $\alpha$ in dome-stage embryos for wt (n = 10), Opto-Control (CRY2 alone, *n* = 4), and Opto-RhoGEF in light (n = 6) and dark (n = 3). (**d-g**) (top) Plots of quantifications of P-MLC (d), F-actin (e), α-catenin (f) and E-cadherin (g) distribution at the cell-fluid vs. cell-cell interface, and (bottom) exemplary high resolution images for the experimental conditions Opto-Control (CRY2 alone) and Opto-RhoGEF either via immunostaining (d, e, g) or live imaging of a knock-in line for α-catenin-citrine (f) (d: Opto-Control *N* = 3 embryos and *n* = 30 cells, Opto-RhoGEF *N* = 4 embryos and *n* = 40 cells; e: *N* = 3 embryos and *n* = 30 cells per condition; f: *N* = 4 embryos and *n* = 40 cells per condition; g: Opto-Control *N* = 6 embryos and *n* = 60 cells, Opto-RhoGEF *N* = 7 embryos and *n* = 70 cells). **(h)** Schematic diagram of the Opto-zGrad tool, which is composed of an AsLOV2 (LOV2) domain attached to a GFP nanobody (NB) and a domain for proteasomal degradation targeting (E3). **(i)** Exemplary 2D confocal sections of blastoderms from zebrafish embryos from a knock-in line for α-catenin-citrine, either without (Control) or with expression of Opto-zGrad. The 90 min timelapse of the illumination corresponds to the stages of sphere until 50% epiboly, with doming at t = 18 min. Membranes labeled with α-catenin-citrine, interstitial fluid with dextran-647. **(j)** Plot of the quantification of α-catenin-citrine fluorescence intensity in control and Opto-zGrad embryos shown in (i) over time (N = 3 embryos per condition). **(k)** Plot of the quantification of relative surface tension $\alpha$ over time for the conditions shown in (i) (n = 140-210 angles per time point; embryo numbers: *N* = 4 Control, *N* = 3 Opto-zGrad). **(l-o)** (top) Plots of quantifications of P-MLC (l), F-actin (m), α-catenin (n) and E-cadherin (o) distribution at the cell-fluid vs. cell-cell interface, and (bottom) exemplary high-magnification images for the experimental conditions *wt* shield-stage and Opto-zGrad shield stage, either via immunostaining (l, m, o) or live imaging of a knock-in line for α-catenin-citrine (n) (l: *N* = 3 embryos and *n* = 30 cells per condition; m: *N* = 5 embryos and *n* = 50 cells per condition; n: *N* = 3 embryos and *n* = 30 cells per condition; o: *N* = 8 embryos and *n* = 80 cells per condition). Yellow and white and arrowheads indicate protein enrichment or reduction at the cell-cell interface, respectively. Statistics: Kruskal-Wallis with Dunn’s post-hoc test (Bonferroni adjustment) (c’) global *p* ≈ 0.0044, individual comparisons: wt vs. Opto-Control *p* ≈ 0.5716, wt vs. Opto-RhoGEF in light *p* ≈ 0.0011, wt vs. Opto-RhoGEF in dark *p* ≈ 0.6121, Opto-Control vs. Opto-RhoGEF in light *p* ≈ 0.0105, Opto-RhoGEF in light vs. Opto-RhoGEF in dark *p* ≈ 0.0201; two-sided Mann-Whitney U-test (d) *p* ≈ 0.0017, (g) *p* ≈ 0.3075, (m) *p* < 0.0001, (o) *p* ≈ 0.0074; two-sided Welch’s t-test (f) *p* ≈ 0.0217; two-sided Student’s t-test (e) *p* < 0.0001, (l) *p* < 0.0001, (n) *p* ≈ 0.0059. Scale bars: 50 µm (c), 30 µm (b, i), 10 µm (d-g, l-o). Source data

To increase ${\gamma }_{{\rm{cf}}}$, we expressed an opto-RhoGEF system in *wt* dome-stage embryos, comprising a CRY2-RhoGEF fusion and membrane-tagged CIBN, which enables light-induced recruitment of RhoGEF to the plasma membrane^57^ (Extended Data Fig. 4a,b and Extended Data Table 1). This increases P-MLC and F-actin levels all along the cell surface (Fig. 3b and Extended Data Fig. 4d,e (yellow arrowheads)). The increased actomyosin contractility at the cell–cell interface further enriches α-catenin at the contact^58^ (Fig. 3b and Extended Data Fig. 4f,g (yellow arrowheads)), suggesting that cell–cell adhesion strength is enhanced. Quantification of the relative surface tension confirmed that the optogenetic control of contractility is sufficient to decrease $\alpha$ below the critical point. This was a specific effect of RhoGEF recruitment, as this is not observed in CRY2 alone or dark conditions (Fig. 3c and Extended Data Figs. 3b and 4c,c′). As a result, cell–cell contacts are stabilized (Extended Data Fig. 3e), leading to spatiotemporally controlled rigidification of dome-stage embryos (Fig. 3c,d and Extended Data Fig. 3c,d).

To increase ${\gamma }_{{\rm{cc}}}$, we generated the opto-zGrad tool, a GFP-targeting nanobody with an AsLOV2 domain and an E3-ligase binding domain, enabling light-induced proteasomal degradation^59,60^ (Extended Data Fig. 4h). When expressed in wt 50% epiboly-stage embryos with endogenously citrine-tagged α-catenin^61^, light exposure triggers the degradation of α-catenin (Fig. 3b (right, white arrowhead), Extended Data Fig. 4i,j, Supplementary Video 1 and Extended Data Table 1). By degrading α-catenin, E-cadherin cannot properly anchor to the actomyosin cytoskeleton^62–64^, disturbing F-actin and P-MLC distribution at the cell–fluid interface (Extended Data Fig. 4l–o). Quantification of the contact surface tension and CLFs confirmed that α-catenin degradation is sufficient to increase $\alpha$ above the critical point, increase CLFs (Fig. 3c, Extended Data Figs. 3e and 4k) and fluidize 50% epiboly-stage embryos with high spatiotemporal precision (Fig. 3c,e and Extended Data Fig. 3c,d).

Given that on contact surface tension manipulations, the cell fraction also changes (Fig. 3c and Extended Data Fig. 3a,b), we uncoupled the two parameters using combinations of optogenetics and osmoregulation. For instance, the opto-RhoGEF-dependent decrease in $\alpha$ also increased the cell fraction (Fig. 3c and Extended Data Fig. 3a), but a combination with mannitol treatment generated tissues occupying the bottom-left regime of the phase diagram (Fig. 3f and Extended Data Fig. 3a,b). Additionally, opto-zGrad-dependent increase in $\alpha$ reduced the cell fraction (Fig. 3c and Extended Data Fig. 3a); however, a combination with ouabain treatment produced tissues occupying the top-right regime of the phase diagram (Fig. 3f and Extended Data Fig. 3a,b). By expanding these manipulations to the marginal region of the blastoderm, which is topologically rigid and highly viscous^5,19^, we were able to start from the pluripotent zebrafish blastoderm, fine-tune the control parameters and engineer it to occupy all the predicted regimes of the phase diagram (Fig. 3g and Extended Data Fig. 3a,b).

For all the engineered tissues, we quantified connectivity and GRC size to map topological rigidity (rigid versus floppy) (Extended Data Fig. 3c,d), and CLFs and/or MPA to map tissue viscosity (solid like versus fluid like) (Fig. 3h,i, Extended Data Figs. 3e and 2d and Supplementary Videos 2 and 3). This enriches the ($\alpha$, $\phi$) morpho-space with genetically identical embryos differing only in their material state and in the underlying control parameters, grouped into four regimes: (1) solid-like, rigid, coupled $\alpha$–$\phi$; (2) solid-like, rigid, uncoupled $\alpha$–$\phi$; (3) fluid-like, rigid, uncoupled $\alpha$–$\phi$; and (4) fluid-like, floppy, coupled $\alpha -\phi$. Thus, solely tuning the relative surface tension and cell fraction in relation to their critical points is sufficient to engineer tissues with accurately controlled material states, allowing us to address how the material state influences the tissue morphogenetic potential.

## Tissue material state discretizes tissue organization states via formation of tricellular contacts

**Fig. 4:**
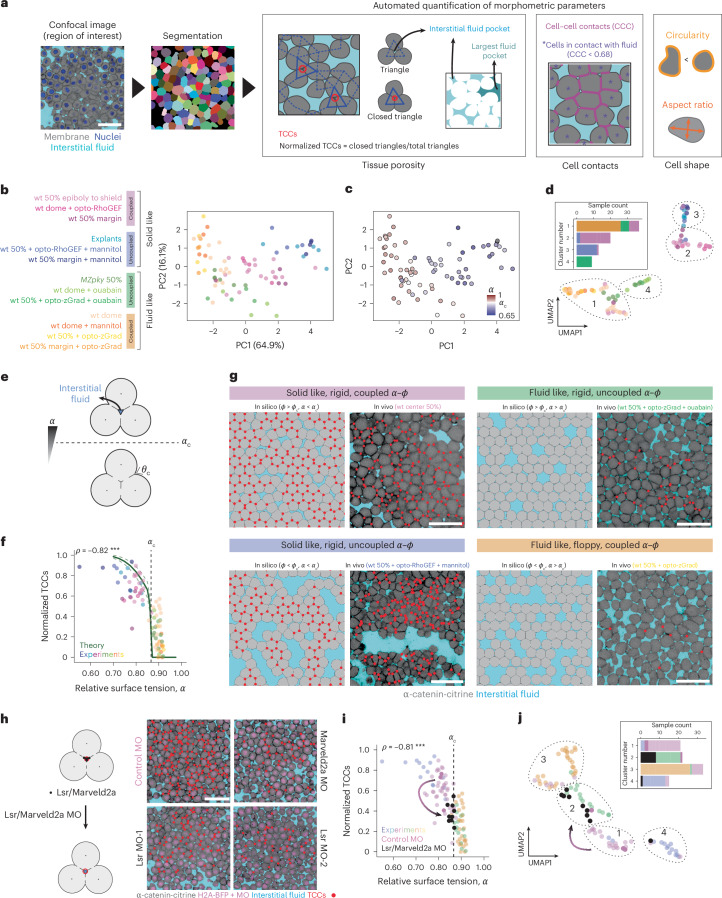
Contact-surface-tension-driven rigidification leads to abrupt formation of TCCs, which dictates tissue morphology and organization. **a**, Schematic of the image analysis pipeline used for cell segmentation and morphometric analysis. **b**, PCA plot of the morphology of tissues occupying the material phase diagram in Fig. 3g. **c**, Same PCA plot as in **b**, but colour coded according to the relative surface tension $\alpha$ values for each tissue. **d**, UMAP plot of the morphology of tissues occupying the material phase diagram in Fig. 3g, with the clusters identified by *k*-means clustering outlined with the dashed lines, and a histogram of the tissue material phase regime composition of each cluster. **e**, Representation of the computation of ${\alpha }_{c}$ at which interstitial spaces between three cells are closed (Supplementary Note III-A). **f**, Simulation at the tissue level for computing the fraction of TCCs that are closed as a function of $\alpha$, with overlaid experimental values from all the tissues shown in **b**. **g**, Example tissue-level simulations (left) and 2D confocal sections of experimental tissues (right) for the four material regimes. The red dots indicate closed TCCs. Membranes labelled with α-catenin-citrine and interstitial fluid with dextran-647. **h**, Left: schematic of TCJ-specific protein (Lsr and Marveld2a) accumulation. Right: representative confocal sections of germ ring-stage control morphants, Marveld2a morphants and Lsr morphants (translational and splicing morpholino). The red dots indicate closed TCCs. Membranes labelled with α-catenin-citrine and interstitial fluid with dextran-647. **i**, Plot of the fraction of closed TCCs as a function of $\alpha$ for the data shown in **f** (transparent dots) and the experimental conditions shown in **h** (dark magenta dots for control morphants and black dots for Lsr/Marveld2a morphants). Embryos per condition: *n* = 3 for control morphants, *n* = 9 for the Lsr/Marveld2a morphants (n = 3 per individual morpholino). The arrow highlights the reduction in the number of closed TCCs. **j**, UMAP plot of the morphology of tissues shown in **d** (transparent dots) together with the experimental conditions shown in **h** (dark magenta dots for control morphants and black dots for Lsr/Marveld2a morphants), with the clusters identified by *k*-means clustering outlined using the dashed lines, and a histogram of the tissue material phase regime composition of each cluster. The arrow highlights the shift in the morphological cluster when TCCs are less able to close. Dashed lines, critical points. Statistics: experimental data *P* < 0.0001 (**f**), *P* < 0.0001 (**i**). Scale bars, 50 µm (**g** and **h**). Source data

**Extended Data Fig. 5:**
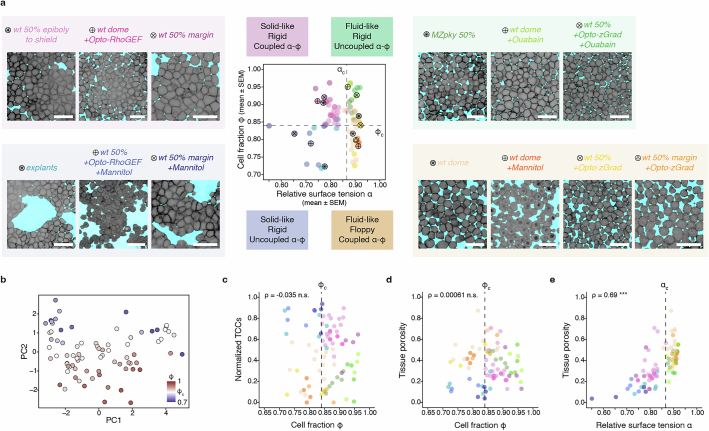
Phase transition-dependent discretization of tissue morphology and organization. **(a)** Exemplary 2D confocal sections showcasing tissue morphology for the experimental conditions occupying each material regime in the phase diagram (embryo numbers as in Fig. 3). Interstitial fluid labeled with dextran-647, membranes with α-catenin-citrine, membrane-GFP, or Lifeact-eGFP. **(b)** Morphospace of all tissues shown in the material phase diagram in (a) plotted against PC1 and PC2, color-coded by cell fraction $\phi$ values for each tissue. **(c)** Plot of the fraction of TCCs that are closed as a function of cell fraction $\phi$ for all the tissues shown in (a). (**d, e**) Plots of tissue porosity as a function of cell fraction $\phi$ (d) and relative surface tension $\alpha$ (e). Dashed lines: critical points. Statistics: ρ: Spearman correlation (c) *p* ≈ 0.76, (d) *p* = 1, (e) *p* < 0.0001. Scale bars: 50 µm (a). Source data

To assess the effects on morphogenesis, we first quantified the morphology of the engineered tissues shown in Fig. 3g using a custom-made image analysis pipeline (Fig. 4a). We then performed dimensionality reduction via principal component analysis (PCA) on eight morpho-features associated with cell shape, cell surface and extracellular space, excluding the control and order parameters of the tissue material state ($\alpha$, $\phi$, <$k$>, GRC, CLFs). Tissues were represented as points in a low-dimensional morpho-space, with axes defined by the PCs (Fig. 4b). PC1 and PC2 accounted for 64.9% and 16.1% of the variance, respectively, and fluid-like and solid-like tissues showed minimal overlap. Analysing the relationship between the PCs and the control parameters revealed that PC1 changes as a function of $\alpha$, whereas PC2 varies with $\phi$ (Fig. 4c and Extended Data Fig. 5b), suggesting that the tissue material state and the control parameters used drive the major morphological differences in tissue organization.

Although tissues belonging to the same material regime exhibit a continuum of $\alpha$ and $\phi$ values, at the morphological level, they appear to exhibit distinct tissue architectures depending on their material regime (Extended Data Fig. 5a). We applied uniform manifold approximation and projection (UMAP) to our dataset^65^, followed by *k*-means clustering to group the datapoints based on similarities in their morpho-features^66,67^. Intriguingly, the four morphological clusters correlate with the four material regimes (Fig. 4d). Clusters 1 and 4 consist almost exclusively of data points corresponding to fluid-like tissues ($\alpha > {\alpha }_{c}$) and clusters 2 and 3 of data points corresponding to solid-like tissues ($\alpha < {\alpha }_{c}$). Additionally, there is a separation between the coupled versus uncoupled regimes (fluid-like tissues: cluster 1, coupled $\alpha$–$\phi$; cluster 4, uncoupled $\alpha$–$\phi$; solid-like tissues: cluster 2, coupled $\alpha$–$\phi$; cluster 3, uncoupled $\alpha$–$\phi$). This suggests that the critical points defining the material regimes are also defining the limits of their morphological regimes, discretizing the potential tissue organization states a tissue can exhibit.

To identify the key dimension accounting for the major morphological differences, we examined which morpho-features primarily contribute to the PCs and found that PC1 is strongly correlated with features related to tricellular contacts (TCCs). In agreement with previous work^18,68^, we observe that in triangular cell motifs, the central interstitial space vanishes and the cells form a TCC depending on the relative surface tension (Fig. 4e). Using the Young–Dupré relation (Supplementary Fig. 5), we show that TCC formation occurs exactly at the critical point ${\alpha }_{c}\approx 0.866$, for which we demonstrated that tissue rigidification takes place. Similarly, simulations using arbitrarily disordered cell packings exhibit a sharp increase in TCC number at ${\alpha }_{c}$ (Supplementary Fig. 6 and Fig. 4f,g). This shows that at ${\alpha }_{c}$, a rigidity transition and a transition from a porous to non-porous regime occur simultaneously, with the tissues acquiring the potential to become fully confluent (Supplementary Fig. 7). TCC quantification in our experimental data revealed that solid-like tissues ($\alpha < {\alpha }_{c}$) display a high number of TCCs, whereas fluid-like tissues ($\alpha > {\alpha }_{c}$) display very few TCCs, independently of their cell fraction (Fig. 4f,g and Extended Data Fig. 5c). Similarly, tissue porosity changes drastically at ${\alpha }_{c}$, but not at ${\phi }_{c}$ (Extended Data Fig. 5d,e), demonstrating that adhesion-driven rigidity transitions are coupled to porosity transitions. These results raise the hypothesis that TCC formation constitutes the cellular mechanism by which adhesion-driven rigidity transitions dictate tissue (re)organization.

**Extended Data Fig. 6:**
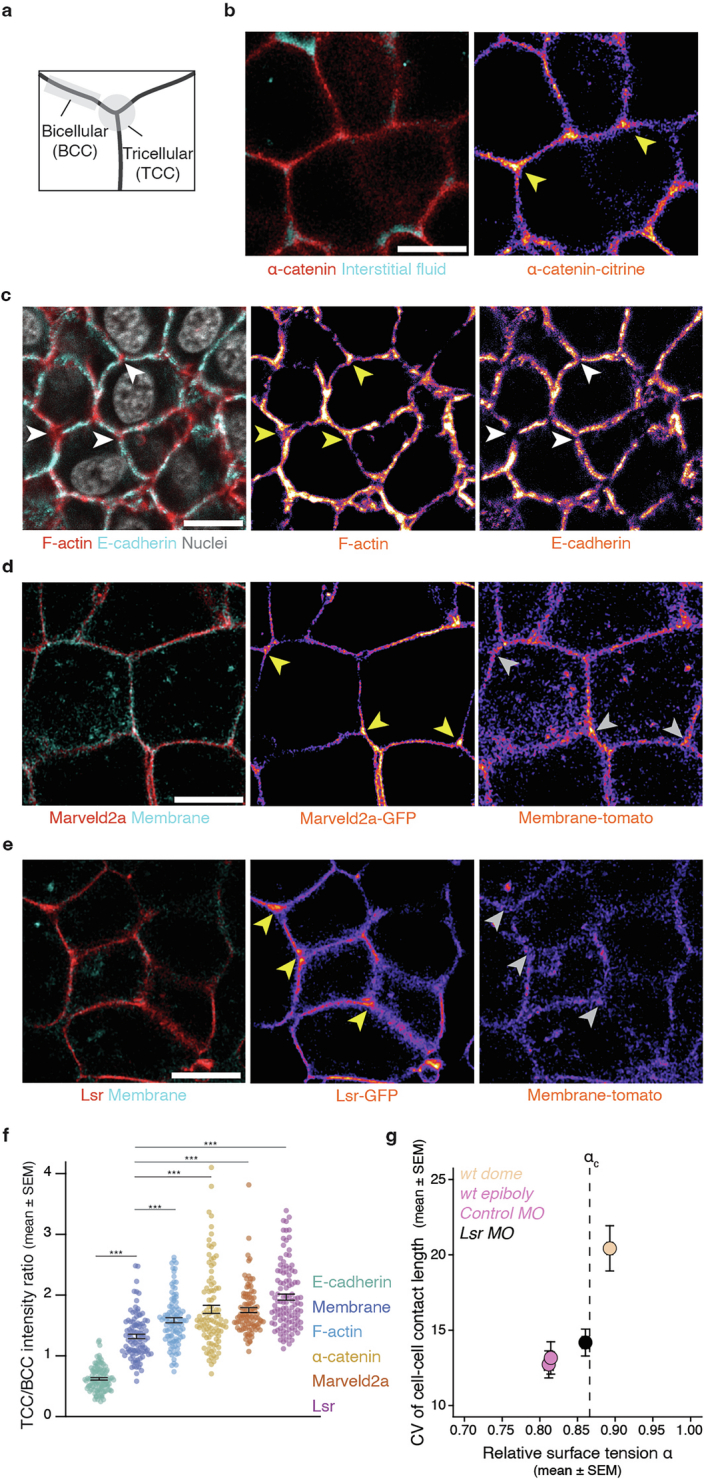
Accumulation of tricellular junction-specific proteins at the tricellular contacts of pluripotent cells undergoing an adhesion-driven tissue rigidification. **a)** Scheme of the quantification of fluorescent protein intensity at the tricellular (TCC) vs. the bicellular (BCC) contacts. **(b-e)** Exemplary high-resolution 2D confocal sections of *wt* shield-stage embryos showing: (b) live imaging of endogenously tagged α-catenin with interstitial fluid labeling using dextran-647; (c) fixed samples stained for F-actin (phalloidin) and E-cadherin; (d) live imaging of tagged Marveld2a and membrane; and (e) Lsr and membrane marker. **(f)** Plot of the quantification of fluorescent protein intensity at the tricellular (TCC) vs. the bicellular (BCC) contacts for the proteins shown in (b-e) (N = 4 embryos each, cells per staining: E-cadherin *n* = 106, Membrane *n* = 100, F-actin *n* = 101, α-catenin *n* = 100, Marveld2a *n* = 100, Lsr *n* = 111). **(g)** Plot of the coefficient of variation in cell-cell contact length as a function of relative surface tension $\alpha$ (embryo numbers for relative surface tension $\alpha$ as in Fig. 4i; embryo number N and contact number n for cell-cell contact lengths: *wt* dome *N* = 8 and *n* = 39, *wt* epiboly *N* = 13 and *n* = 45, control MO *N* = 6 and *n* = 30, Lsr MO *N* = 8, *n* = 39). Gray arrowheads indicate TCCs, and yellow and white arrowheads indicate protein enrichment or depletion at the TCCs, respectively. Statistics: One-way ANOVA (f) global *p* < 0.0001, individual comparisons: F-actin *p* = 0.0001, all others *p* < 0.0001. Scale bars: 10 µm (b-e). Source data

To test this, we examined tissue architecture in tissues undergoing adhesion-driven rigidification but impaired in tricellular gap closure. We first explored if the TCCs resemble tricellular junctions (TCJs) observed in epithelia, which are specialized high-tension structures formed by specific proteins such as Tricellulin/MARVELD2 and Angulin-1/LSR^69–72^. Live imaging of TCJ proteins shows that tissues undergoing adhesion-driven rigidification accumulate the TCJ-specific proteins Marveld2a and Lsr and the mechanosensitive proteins F-actin and α-catenin, but are depleted of E-cadherin (Extended Data Fig. 6a–f). On the basis of this molecular composition, we downregulated Marveld2a and Lsr expression, which reduced TCJ formation while maintaining a solid-like tissue state ($\alpha < \alpha$_c_, low CLFs) (Fig. 4h,i and Extended Data Fig. 6g) and, subsequently, quantified the tissue morphology. These tissues shifted from a morphology characteristic of the coupled $\alpha$–$\phi$, rigid and solid-like regime towards that of tissues occupying the uncoupled $\alpha$–$\phi$, rigid and fluid-like regime (Fig. 4j, magenta versus black points). These findings show that adhesion-driven rigidification enforces tricellular gap closure and porosity loss, which substantially shifts tissue organization, uncovering that the mechanism by which the tissue material state dictates tissue morphology and organization is via the formation of TCJs.

## Adhesion-driven solidification triggers lumenogenesis and apical polarity in unjammed pluripotent tissues

**Fig. 5:**
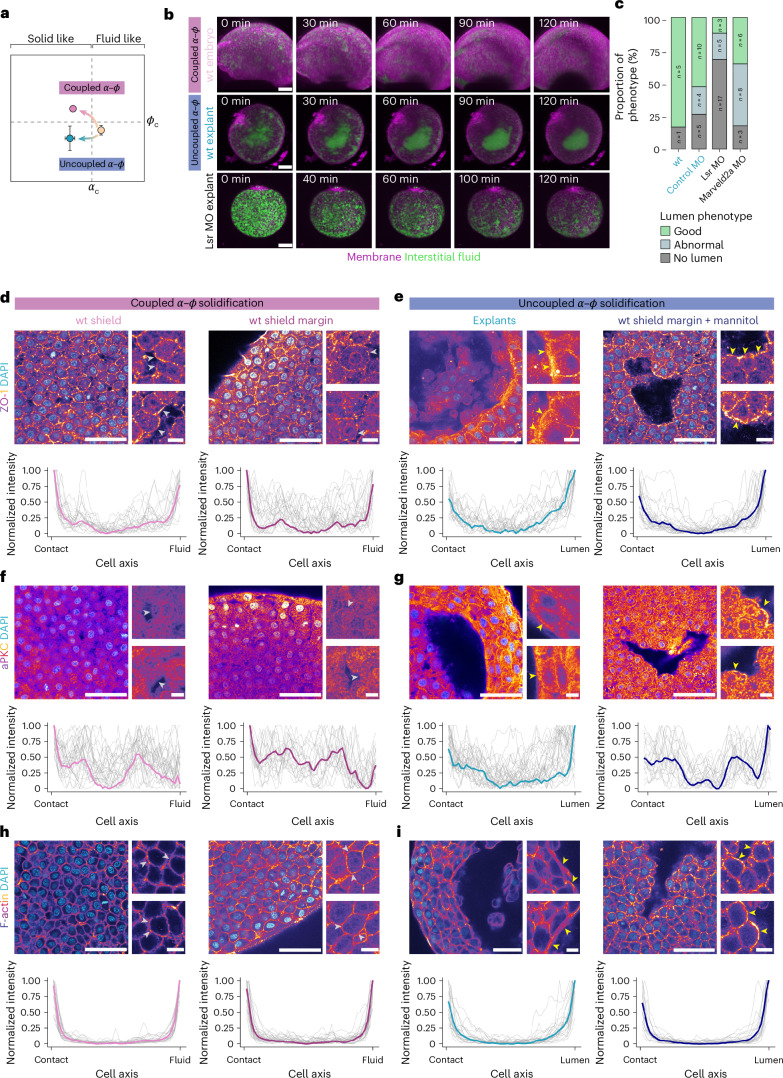
Uncoupling density- from adhesion-dependent solidification in pluripotent tissues triggers the acquisition of epithelial polarity. The data shown here are related to Supplementary Videos 4–10. **a**, Phase diagram of the tissue material regimes as a function of cell fraction $\phi$ and relative surface tension $\alpha$, showcasing tissue solidification by coupling (magenta) versus uncoupling (blue) the two control parameters. **b**, 3D projections of light-sheet imaging (20–40-min intervals shown) of coupled (wt centre) versus uncoupled (wt explants) tissue solidification versus explants from Lsr morphant embryos with defective closure of TCCs, showing lumen formation only in the uncoupled regime with closed TCCs. **c**, Plot of the qualitative assessment of lumen formation in explants of the conditions shown in **b** (explant numbers per condition: wt *n* = 6, control MO *n* = 19, Lsr MO *n* = 25, Marveld2a MO *n* = 17). **d**–**h**, Example confocal sections of embryonic tissues undergoing coupled (experimental conditions: left, wt shield centre; right, wt shield margin) (**d**, **f** and **h**) versus uncoupled (experimental conditions: left, explants; right, wt shield margin + mannitol) (**e**, **g** and **i**) rigidity transitions immunostained for ZO-1 (**d** and **e**), aPKC (**f** and **g**) and F-actin (phalloidin) (**h** and **i**). For each condition, a representative large-scale view of the tissue is shown (left), together with two magnified regions (right) highlighting cell–fluid interfaces (arrowheads). Bottom: intracellular distribution of the indicated proteins along the axis from cell–cell contact to cell–fluid/lumen interface, showing the mean profile across samples (thick coloured line) and the corresponding profiles from individual cells (thin grey lines). Quantified number of embryos *N* and total number of cells *n* per staining and condition: ZO-1 wt shield centre *N* = 3, *n* = 21, wt shield margin *N* = 4, *n* = 24, explants *N* = 4, *n* = 21, wt shield margin + mannitol *N* = 4, *n* = 26; aPKC wt shield centre *N* = 4, *n* = 28, wt shield margin *N* = 3, *n* = 21, explants *N* = 9, *n* = 29, wt shield margin + mannitol *N* = 3, *n* = 21; F-actin wt shield centre *N* = 3, *n* = 21, wt shield margin *N* = 4, *n* = 26, explants *N* = 5, *n* = 21, wt shield margin + mannitol *N* = 4, *n* = 21. The yellow arrowheads indicate protein apical enrichment. Scale bars, 100 µm (**b**); 50 µm (overviews in **d**–**i**); 10 µm (magnified regions in **d**–**i**). Source data

The closure of tricellular gaps and the concomitant loss of tissue porosity during solidification are reminiscent of epithelialization, raising the question of whether pluripotent tissues could tune their epithelial fate alongside their material state. To investigate this, we examined differences in tissue organization and epithelial characteristics during coupled versus uncoupled solidification (Fig. 5a). The 3D time-lapse images revealed that although in both cases, TCJs form and interstitial fluid is locally excluded between the cells, during the coupled transition, the interstitial fluid is redistributed into small pockets in regions in which tricellular gaps have not yet closed (Fig. 5b and Supplementary Video 5). By contrast, during uncoupled solidification, the interstitial fluid accumulates in larger pockets that eventually coalesce into bigger lumen-like structures (Fig. 5b, Extended Data Fig. 5a and Supplementary Videos 4, 6 and 7). In particular, the formation of lumen-like structures depends on TCJ closure, since it is severely compromised upon TCJ protein downregulation (Fig. 5b,c and Supplementary Videos 8–10).

**Extended Data Fig. 7:**
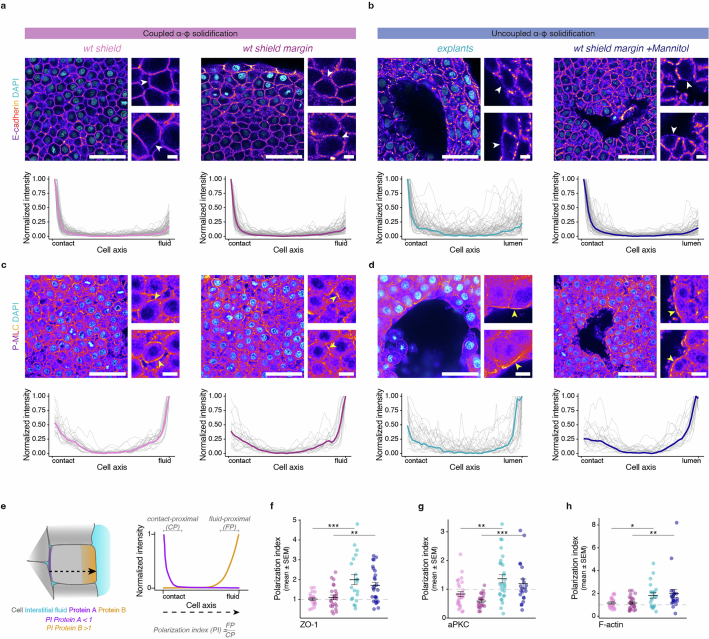
Quantification of cell polarity markers in coupled vs. uncoupled tissue rigidification. **(a-d)** Exemplary confocal sections of embryonic tissues undergoing coupled (experimental conditions left: *wt shield center*, right: *wt shield margin*) (a, c) vs. uncoupled (experimental conditions left: *explants*, right: *wt shield margin +Mannitol*) (b, d) rigidity transitions immunostained for E-cadherin (a, b) and P-MLC (c, d). For each condition, a representative large-scale view of the tissue is shown (left panel), together with two magnified regions (right panels) highlighting cell-fluid interfaces (arrowheads). The bottom panel displays a plot of the intracellular distribution of the indicated proteins along the axis from cell-cell contact to cell-fluid/lumen interface, showing both the mean profile across samples (thick colored line) and the corresponding profiles from individual cells (thin gray lines). Quantified number of embryos N and total number of cells n per staining and condition: E-cadherin *wt shield center* *N* = 10, *n* = 70, *wt shield margin* *N* = 9, *n* = 61, *explants* *N* = 13, *n* = 52, *wt shield margin +Mannitol* *N* = 10, *n* = 63; P-MLC *wt shield center* *N* = 3, *n* = 21, *wt shield margin* *N* = 3, *n* = 21, *explants* *N* = 4, *n* = 24, *wt shield margin +Mannitol* *N* = 3, *n* = 21). Yellow arrowheads indicate protein apical enrichment. **(e)** Schematic diagram of the quantification method of the protein intracellular distribution using histogram profiles along a cell axis spanning from a cell-cell contact to the opposite cell-free surface. **(f-h)** Plots of the polarization index for ZO-1 (f), aPKC (g) and F-actin (h), calculated as the ratio of normalized fluorescence intensity at the region proximal to the fluid vs. the region proximal to the opposite contact from the protein distribution histograms shown in Fig. 5d-I (numbers as in Fig. 5d-i). Points represent individual cells, lines the mean of all cells. Statistcs: Two-sided Welch’s t-tests (f) *wt shield center* vs. *explants p* ≈ 0.0010*, wt shield margin* vs. *wt shield margin +Mannitol p* ≈ 0.0053, (h) *wt shield center* vs. *explants p* ≈ 0.0168; two-sided Mann-Whitney U-test (g) *wt shield center* vs. *explants p* ≈ 0.0042*, wt shield margin* vs. *wt shield margin +Mannitol p* ≈ 0.0004, (h) *wt shield margin* vs. *wt shield margin +Mannitol p* ≈ 0.0077. Scale bars: 50 µm in the overviews in (a-d), 10 µm in the magnified regions in (a-d). Source data

Last, we explored whether lumens are accompanied by molecular signatures of epithelia, such as the acquisition of apical polarity^73^. Strikingly, immunostaining for polarity markers revealed that uncoupled solidification not only induced morphological lumenogenesis but also promotes apical polarization, as observed by the depletion of E-cadherin (Extended Data Fig. 7a,b) and the enrichment of F-actin, ZO-1, aPKC and P-MLC at the lumen interface (Fig. 5d–i and Extended Data Fig. 7c–h). This shows that adhesion-driven solidification primes epithelial-like character via TCJ formation and loss of porosity. However, when the transition occurs by uncoupling changes in contact surface tension and cell fraction, it further triggers epithelial tissue organization via lumen formation and acquisition of apical polarity at the molecular level.

## Outlook

This work shows that the (un)coupling of multiple microscopic control parameters in living materials not only determines their macroscopic material state but also their morphogenetic potential. We identify contact surface tensions as a parameter linking the material state transitions to tissue organization and reveal the underlying cellular mechanism: at a critical point in relative surface tensions, the tissue solidifies and TCCs close, abruptly reducing the tissue porosity and initiating an epithelial-like organization.

We focused on the roles of density and adhesion, proposing a phase diagram for living tissues deviating from the classic diagrams of inert materials^33^. In non-confluent tissues, solidification results from an adhesion-driven and not density-driven rigidity transition, which reduces cell–cell contact dynamics. This is consistent with previous experimental and computational studies, showing that reducing adhesion promotes tissue fluidization^9,18,74^. However, theoretical predictions from vertex models can be interpreted that increasing cell–cell adhesion in confluent tissues induces fluidization^17,75^. This discrepancy probably stems from the model assumption of a preferred cell perimeter and an adhesion formulation in which more adhesion results by default in larger contact interfaces^17^. Moreover, in the vertex models, confluency is imposed as a hardcore external constraint, irrespective of the cell properties, whereas in our framework, the transition to confluency is reversible, with porosity and the cell–fluid interface reappearing if the contact surface tension increases. This highlights a fundamental difference between the theoretical approximation of non-confluent and confluent tissues. Moreover, these two tissue configurations differ in the localization of AJ and actomyosin components, which colocalize at cell–cell interfaces in confluent tissues but segregate between the cell–cell versus cell–fluid interfaces in non-confluent tissues (Fig. 3b)^5,40,41,51–53^. This motivates future theoretical and experimental work to explore how the spatial organization of AJ complex molecular components affects the tissue material state.

Here, by taking advantage of the spatial cellular organization of the AJ complex, we uncoupled adhesion-driven versus density-driven rigidification in the pluripotent embryo and uncovered a fundamental role of tissue phase transitions in tissue organization. The control parameter uncoupling initiates an epithelialization process in which the concomitant closure of TCCs and loss of tissue porosity at ${\alpha }_{c}$ lead to lumenogenesis and apical cell polarity. This resembles the acquisition of epithelial character during epithelial-to-mesenchymal transitions, which is often accompanied by lumen formation and cell polarization during embryogenesis and organogenesis (for example, blastocoel^76,77^, neural tube^78^, Kupffer’s vesicle^79^, kidney tubules and mammary gland^80^). Epithelial cell states are not absolute, but exist as dynamic regimes in which ranges of epithelial and mesenchymal features coincide^81,82^. Our finding that the control parameter coupling defines the degree of epithelial character a tissue acquires raises the possibility that this coupling contributes to the diversity of epithelial organization states a tissue can exhibit. Last, tissue physical properties can influence biochemical signalling^83–86^ and cellular behaviour^87,88^, suggesting that via phase transitions, tissue physical changes are coordinated with the underlying molecular and cellular dynamics.

We conclude that phase transitions play unexpected roles in developing systems, extending beyond tissue deformability^89^, originating from the ability of living materials to operate near multiple critical points^90^, which may act as a mechanism channelling the possible morphogenetic trajectories of developing tissues.

## Methods

### Zebrafish handling

Zebrafish (*Danio rerio*) were raised at 28.5 °C under a 14-h light/10-h dark cycle^91^. The following zebrafish strains were used in this study: wt AB, *MZpky* (ref. ^46^), *Gt(ctnna-citrine)*^*ct3a*^ (ref. ^61^), *Tg(actb2:Lyn-Tomato)* (ref. ^92^) and *Tg(actb2:Lifeact-eGFP)* (ref. ^93^). Zebrafish embryos were maintained at 28.5 °C in 1× Danieau’s medium. All animal experiments were carried out according to the guidelines of the Committee for Animal Welfare and Institutional Animal Care and Use under EMBL’s Policy on the Protection and Welfare of Animals Used for Scientific purposes (IACUC code 21-010_HD_NP).

### Cloning

The pCS2(+) vector was used for cloning and mRNA production in all experiments. The opto-zGrad plasmid was generated by combining zGrad^59^ and opto-nanobody^60^ systems. PCR-amplified *short AsLOV2* (Addgene plasmid number 159592) and *zGrad* (Addgene plasmid number 119716) were inserted into pCS2(+) via Gibson assembly. *AsLOV2* was inserted into the *VHHGFP4* nanobody (used for zGrad; EBI entry) at the outer loop position, as in ref. ^60^. For the opto-RhoGEF tool, opto-RhoAGEF2-mCherry and opto-RhoAGEF2 were subcloned from the ARHGEF11(DHPH)-CRY2-mCherry construct (Addgene plasmid number 89481) into pCS2(+) via Gibson assembly. All CIBN constructs, including CIBN-mCherry-CAAX and CIBN-CAAX, were derived from existing constructs in the de Renzis’s laboratory^94^. To visualize TCJs, Lsr and Marveld2a were selected based on their expression in early developmental stages^95,96^. cDNA of lsr-201 and marveld2a-202 was cloned from 24-hpf larvae using TRIzol LS RNA extraction and SuperScript III cDNA synthesis (Invitrogen). The cloned fragments were inserted into a pCR 2.1-TOPO TA vector using the TOPO TA Cloning Kit (Invitrogen, category number 450641). Lsr-GFP and Marveld2a-GFP constructs were subsequently generated via Gibson assembly. The sequences of all oligos used for cloning are provided in the Supplementary Table.

### Embryo microinjections and explant formation

Zebrafish embryos were injected using glass capillary needles (30-0020, Harvard Apparatus) made with a P-97 needle puller (Sutter Instrument) and attached to a PV820 microinjector system (World Precision Instruments). mRNA microinjections were performed at the one-cell stage into the yolk or at the two-cell stage into one of the cells to generate mosaics. mRNAs were synthesized from linearized plasmids using the mMESSAGE mMACHINE SP6 Transcription Kit (Invitrogen, catalog number AM1340). The following mRNAs were injected: (1) cell membrane labelling: 50–70-pg *membrane-RFP* (ref. ^97^), *membrane-GFP* (ref. ^98^); (2) nuclear labelling: 20–40-pg *H2A-BFP* (ref. ^99^), *H2A-mCherry* (ref. ^100^) or *H2B-GFP* (ref. ^101^); (3) TCJ labelling: 17.5-pg *lsr-GFP* and 17.5-pg *marveld2a-GFP*; (4) α-catenin-citrine degradation: *Gt(ctnna-citrine)*^*ct3a*^ homozygous embryos were microinjected with 35-pg *opto-zGrad* and co-injected with a nuclear or cell membrane marker; (5) RhoGEF activation to decrease relative surface tension and its control: 50–70-pg *CIBN-CAAX* or *CIBN-mCherry-CAAX* co-injected with 17.5-pg *opto-RhoGEF-CRY2* or *CRY2* alone. These amounts were halved to generate mosaic embryos.

Morpholinos targeting *lsr* and *marveld2a* and a standard control morpholino were purchased from Gene Tools (https://store.gene-tools.com) and co-injected with labelled histone mRNA into one-cell stage embryos, as detailed in the Supplementary Table.

To label the interstitial fluid, high-stage dechorionated embryos were microinjected with 0.5 nl of 0.6 µg µl^−1^ of dextran-Alexa Fluor 647 (10,000 MW; Invitrogen, catalog number D22914) or dextran-Alexa Fluor 488 (10,000 MW; Thermo Fisher, catalog number D22910) into the blastoderm interstitial fluid space. Explants were prepared by isolating the blastoderm from high-stage embryos and maintained in 1× Danieau’s medium. Stage was determined relative to their sibling control embryos. Interstitial fluid labelling of explants was performed by microinjection 30 min after blastoderm excision, allowing tissue rounding and wound healing.

### Pharmacological treatments

To increase the interstitial fluid fraction, high-stage dechorionated embryos were injected into the blastoderm interstitial space with 0.5 nl of 400-mM D-mannitol (Sigma-Aldrich, M4125-100G) plus 0.6 µg µl^−1^ of dextran-Alexa Fluor 647 to label the interstitial fluid. To decrease the interstitial fluid, dechorionated two-cell stage embryos were incubated with 1-mM ouabain (Sigma-Aldrich, 4995-1GM) until sphere stage, followed by interstitial fluid labelling.

### MPA and viscosity measurements

Blastoderm viscosity was measured by MPA based on previously established protocols^5,19,24^. Briefly, embryos were placed on 3% methylcellulose-coated glass coverslips in 1× Danieau’s solution on an inverted Leica SP5 microscope. A fire-polished, passivated (with heat-inactivated FBS) micropipette of 35-mm inner diameter, 30° bent, with a spike end (Biomedical Instruments) was inserted into the blastoderm just below the EVL. Micropipette movements were controlled by motorized micromanipulators (Eppendorf Transferman, Nk2). On pipette insertion, an aspiration pressure of 150 Pa was immediately applied using a Microfluidic Flow Control System Pump (Fluigent, Fluiwell) (negative pressure ranging from 7 to 750 Pa, pressure accuracy of 7 Pa, change rate of 200 Pa s^−1^) and Dikeria micromanipulation software. The value of the applied pressure (150 Pa) was set according to prior test aspiration experiments, and increased in a stepwise process (10 Pa/20 s) until the aspirated tissue started flowing into the pipette. Pressure was applied until the tissue flowed into the pipette at a constant velocity and then immediately released. Images to monitor tissue aspiration and relaxation were acquired every 500 ms. Viscosity calculations were performed as previously described^5,19,24^, using a customized Fiji macro to calculate changes in the tongue length during aspiration and relaxation over time. The slope of the aspiration curve (${L}_{{\rm{asp}}}$) at the point of constant flow depends on the viscosity $\eta$, ${L}_{{\rm{asp}}}={R}_{{\rm{p}}}(\Delta {\rm{p}}-{{\rm{p}}}_{{\rm{c}}})/3{\rm{\pi }}\eta$, where ${R}_{p}$ is the radius of the pipette, $\Delta P$ is the applied pressure and ${P}_{c}$ is the critical pressure. During the relaxation, the tissue retracts at a velocity ${L}_{{\rm{ret}}}={R}_{p}\left({P}_{c}\right)/3\pi \eta$. From the aspiration and retraction rates, viscosity can be calculated as $\eta ={R}_{{\rm{p}}}\Delta {\rm{p}}/3{\rm{\pi }}({L}_{{\rm{asp}}}+{L}_{{\rm{ret}}})$. The wt, explant and *MZpky* viscosity data were reused from refs. ^5,19^.

### Immunostaining

Samples were fixed in Glyoxal solution mix. For a 4-ml fixative preparation, 2.835 ml of H_2_O, 0.789 ml of 100% EtOH, 0.313 ml of Glyoxal (40% stock solution; Sigma-Aldrich, 128465) and 30 µl of acetic acid (100%) were mixed; pH was adjusted to 4–5 with 1-M NaOH based on ref. ^102^. Dechorionated zebrafish embryos and explants were incubated in Glyoxal solution mix for 1 h (30 min on ice and 30 min at room temperature), followed by 15 min of quenching in 100-mM NH_4_Cl/100-mM glycine. Explants were pre-permeabilized for 2 h in 1× phosphate-buffered saline (PBS)/1% Triton X-100/2% bovine serum albumin, followed by overnight incubation in blocking/permeabilizing solution (B/PS; 1× PBS/0.5% Triton X-100/2% bovine serum albumin) for both explants and embryos. Primary antibodies were incubated overnight at 4 °C in B/PS: mouse anti-E-cadherin (BD Biosciences, catalog number 610181, 1:200), mouse anti-ZO-1 (Thermo Fisher Scientific, 33-9100, 1:100), rabbit anti-P-MLC 2 (Ser19) (Cell Signaling, 3671, 1:100), rabbit anti-pan-cadherin (Sigma-Aldrich, C3678, 1:200) and rabbit anti-aPKC (Santa Cruz Biotechnology, sc-216-G, 1:200). After three washes in B/PS (30 min each), samples were incubated at 4 °C with secondary antibodies in B/PS overnight or for 2 h at room temperature. The following secondary antibodies were used: goat Alexa Fluor 546 anti-rabbit (Thermo Fisher Scientific, A11010, 1:500), goat Alexa Fluor 488 anti-rabbit (Thermo Fisher Scientific, A11008, 1:500), goat Alexa Fluor 546 anti-mouse (Thermo Fisher Scientific, A11003, 1:500), goat Alexa Fluor 488 anti-mouse (Thermo Fisher Scientific, A11001, 1:500). Finally, samples were washed in 1× PBS/0.5% Triton X-100, followed by 30-min incubation in 300-nM DAPI/1× PBS staining solution (Invitrogen, D1306) in PBS with 1× rhodamine-phalloidin (Abcam, ab235138) or Alexa Fluor 647 phalloidin (Abcam, ab176759), if required. After a final wash with PBS, the samples were mounted in 80% glycerol/0.4% *N*-propyl-gallate/1× PBS.

### Image acquisition

Dechorionated embryos and explants were embedded in 0.6% low-melting-point agarose (Invitrogen, catalog number 16,520-050) on a customized agarose mould in a 60-mm dish (Greiner, 628102). Mounted live samples were kept at 28.5 °C throughout acquisition. Live imaging was performed on an upright ZEISS LSM 980 with Airyscan 2 with Axio Examiner. Confocal live imaging of deep cells to reconstruct connectivity maps was performed with a W Plan-Apochromat ×20/1.0 Corr DIC M27 75-mm objective. Immunostaining samples were imaged with a LD LCI Plan-Apochromat ×40/1.2 Imm Korr DIC M27 ZEISS objective both in confocal and fast Airyscan super-resolution modules. CLFs were imaged with an inverted ZEISS LSM 980 with Airyscan 2 (MPLX SR-8Y mode) and C-Apochromat ×40/1.2-W autocorr FCS M27 objective or with an upright ZEISS LSM 980 with Airyscan 2 (MPLX SR-4Y mode) and W Plan-Apochromat ×20/1.0 Corr DIC M27 75-mm objective. Time-lapse images were acquired over at least 10 min in 10-s intervals. Images were acquired in ZEN3.3 (blue edition) software (Carl Zeiss) and processed using Fiji (https://fiji.sc/). Light-sheet imaging was performed on a Viventis Deep Imaging System with ×10/0.2-numerical-aperture illumination objectives and either 16×/0.8-numerical-aperture or 25×/1.1-numerical-aperture detection objectives, using Viventis Microscope software (v2.1.0.2). Embryos and explants were mounted in 0.6% low-melting-point agarose (Invitrogen, catalog number 16,520-050) in Viventis Deep sample holders (Single Well Pocket 850, 20533021, and Single Well Pocket 1000, 20533022), and four-dimensional datasets were acquired as *z* stacks with a step size of 2–4 µm at 15- or 20-min intervals.

### Optogenetics

Embryos were kept in the dark until the selected developmental stage. To prevent photoactivation, all sample handling and mounting were performed under red light filters (Lee Filter 106, Primary Red). Pre-imaging photoactivation was performed at the sphere stage in most experiments, using a 50-W/60-Hz blue LED lamp. For imaging, embryos were oriented for transverse and sagittal blastoderm views and imaged/photoactivated from sphere to shield stage. For α-catenin-citrine degradation, *Gt(ctnna-citrine)*^*ct3a*^ homozygous embryos were microinjected with opto-zGrad and photoactivation and imaging of α-catenin-citrine degradation were carried out with 488-nm light pulses (laser power of 6–8%, corresponding to an out-of-objective power of 104–140 µW) every ~3 min, with a stack size of ~45 µm (4-µm spacing). Dark effects were sometimes observed in a concentration-dependent manner. For opto-RhoGEF experiments, *Gt(ctnna-citrine)*^*ct3a*^ embryos microinjected with the opto-RhoGEF system were photoactivated/acquired with a 5%–8% 488-nm laser (corresponding to 86–140 µW) in a total *z* stack of 45 µm (4-µm spacing), with pulses every ~5 min.

### Data analysis and quantification

All acquired data were processed using Fiji^103^ and Cellpose (v. 2 and v. 3)^104,105^. R (v. 4.2.2) was used for data analysis and plotting (ggplot2 v. 3.5.1)^106,107^.

#### Image segmentation

Blastoderm images were segmented using Cellpose (v. 2 and v. 3), with manual corrections where necessary.

#### Reconstruction of connectivity maps and rigidity analysis

Cell connectivity was defined on 2D confocal sections of the second–third deep-cell layer in which cell–cell contacts and interstitial fluid were differentially labelled. The interstitial fluid channel was binarized and, if needed, median-filtered (1–2 px) in Fiji to enhance gaps. Cells within the same focal plane with no interstitial fluid between them were considered as contacting. The non-binarized image was compared for verification. The connectivity networks were reconstructed based on the above-described Cellpose segmentation. Label adjacency was assessed using a custom-made Fiji plug-in using the MorpholibJ function ‘Region Adjacency Graph’^108^. The plug-in allowed labels to be manually added/removed and single links to be manually curated for corrected networks. The plug-in generates a text file with cell centroids (*x*,*y*) for each cell label and a text file for contact adjacency (based on cell labels) used later for rigidity percolation analysis. The coordinates of each cell centroid and their contacting neighbours were identified and plotted using the standard Python package matplotlib.

#### Cell connectivity and tissue rigidity

Cell connectivity was calculated in each confocal section as the total number of contacts (as described in connectivity map reconstruction) divided by the total number of cells. Normalized connectivity (<$k$>) was calculated in each confocal section as connectivity *C* divided by the maximum potential connectivity (${k}_{\max }$) (computed as described in ref. ^19^). The 2D approximation of rigidity can predict 3D tissue material state, since local 3D connectivity analysis showed that they cross the 3D-predicted critical point in rigidity percolation (½ of the maximum potential connectivity)^19,109^. Due to the lack of algorithms to compute GRC size in three dimensions and to extract 3D cellular networks from imaging data, in this study, we focus on the 2D representation. The rigidity analysis to identify floppy and rigid areas, GRC and cluster size distributions was performed on the connectivity maps using a Python version of the pebble algorithm (http://pebble.py).

#### Cell fraction, tissue porosity and lumen formation

Cell fraction $\phi$ was calculated as $\phi =1-{\rm{ff}},$ where ${\rm{ff}}$ is the fluid fraction. ${\rm{ff}}$ was calculated from 2D confocal sections at the second deep-cell layer, where the interstitial fluid channel (labelled with Alexa Fluor 647 dextran) was first converted into a binary image, as described above. A signal intensity histogram analysis of the binary image was performed in Fiji, and ${\rm{ff}}$ was obtained from the average grey value normalized to the maximum signal intensity of the histogram. For simulated tissues, we created a .ps figure from Surface Evolver tiling and segmented the image to identify cells and fluid. The fluid fraction was computed as the ratio of fluid-labelled pixels to the total number of pixels. Tissue porosity was quantified as a function of the number of pockets per cell times the mean cell perimeter fraction in contact with the interstitial fluid.

#### Relative surface tension

Cell–cell contact angles were measured in degrees from two independent 2D confocal sections in Fiji, and converted to radians to calculate relative surface tension $\alpha$.

#### Morpho-feature quantification

Cell and tissue morphological features were extracted from segmented microscopy images using a custom high-throughput Python pipeline. The analysis was performed using ImageIO (v. 2.34.2), scikit-image (v. 0.24.0), NumPy (v. 1.26.4), pandas (v. 2.2.2), and SciPy (v. 1.14.0) in Python (v. 3.12.4). Labelled segmentation masks generated by Cellpose were upscaled 4 × 4 to avoid the loss of small interstitial fluid pockets and processed iteratively using os. Each image was loaded with imageio.imread(), and individual cell labels were extracted using numpy.unique(). Cellular morphological properties—area, perimeter, orientation, major axis length, minor axis length and circularity—were computed using skimage.measure.regionprops(). For TCCs, a junction map was generated using scipy.ndimage.generic_filter(), identifying locations in which three or more cells meet. These values were normalized by the total number of triangles in the network, obtained from the connectivity map described above and the mathematical derivation presented in equation (9) of the Supplementary Information. To analyse cell–cell and cell–fluid contacts, the background (interstitial fluid) of each image was segmented using skimage.measure.label(). Cell labels were dilated by 1 px, and the overlap with neighbouring cells and background regions was calculated, enabling the quantification of cell–cell contact proportions and the identification of the corresponding background regions. The same approach was used to analyse fluid pockets. For each input segmentation, the extracted parameters were summarized as mean and s.e.m. The values for area, perimeter, circularity and adjacent labels were extracted for individual cells and fluid pockets.

#### Dimensionality reduction and clustering

PCA was performed on metrics qualitatively describing cell and tissue morphology (normalized number of TCCs, percentage of closed triangles, mean cell perimeter fraction in cell–cell contacts, fraction of cells in contact with fluid, circularity, maximum fluid pocket size, number of pockets per cell and cell aspect ratio) using the prcomp() function of the R stats package (R (v. 4.2.2) used in all of the analyses), along with centering and scaling to unit variance for normalization. Metrics acting as the control parameters of material state phase transitions (relative surface tension, cell fraction, cell shape index and connectivity) were not included. Tissues were represented in a 2D morpho-space by plotting them against PC1 and PC2 using ggplot2. UMAP was performed on the same scaled metrics using the umap package (v. 0.2.10.0) in R. Multiple combinations of the parameters n_neighbors and min_dist were tested; the values reported here (n_neighbors = 8, min_dist = 0.08 for Fig. 4d; n_neighbors = 12, min_dist = 0.1 for Fig. 4j) were selected for visualization. The overall clustering patterns were qualitatively consistent across parameter choices. The tissues were represented in a 2D morpho-space by plotting them against UMAP1 and UMAP2 using ggplot2. To assign each tissue to a cluster, *k*-means clustering was performed on the UMAP1 and UMAP2 coordinates using the kmeans() function of the R stats package, specifying four cluster centres based on the number of material phase space regimes.

#### Cell–cell CLFs

Cell–cell CLFs were calculated from 2D confocal time series acquired using the Fast Airyscan mode (10-s interval, 10-min time-lapse duration). For each time point, the contact length was measured using the freehand line tool in Fiji. Then, the contact lengths were normalized to the average of the contact lengths during a 10-min duration and the coefficient of variation (CV) for each contact was calculated. The average CV of at least 12 contacts from at least three embryos per time point and the condition was plotted.

#### Cell and nucleus area and aspect ratio

Cell/nucleus area and aspect ratio were measured using Fiji by manually tracing the cell/nucleus outline with the freehand selection tool and extracting area and shape descriptors. Cells/nuclei were quantified across several *z* layers, excluding the dividing cells.

#### Dividing cells

The proportion of dividing cells was quantified in 30-µm *z* stacks of the blastoderm, excluding EVL cells. Dividing versus non-dividing cells were quantified using the Fiji Cell Counter plug-in. Cells were considered dividing until the end of cytokinesis.

#### α-catenin quantification

To quantify α-catenin degradation with opto-zGrad and assess photobleaching, α-catenin-citrine intensity was measured every ~10 min as the mean grey value within a defined region on the sum projection of four consecutive *z* layers (total, 16 µm), starting from the second deep-cell layer, using Fiji. For each embryo, the values were normalized by the respective initial intensity.

#### Protein (sub)cellular distribution quantification

The relative distribution of AJ components and cortical cytoskeleton was quantified as the ratio of signal intensity in the cell–fluid versus cell–cell interface. Signal intensities were measured in Fiji by drawing 5-px-wide (α-catenin-citrine live), 10-px-wide (E-cadherin-YFP live) or 15-px-wide (immunostainings) freehand lines along each membrane compartment, and the mean grey values were extracted. At least ten cells per embryo were analysed across multiple *z* planes. A similar strategy was used to quantify the relative distribution of multicellular junction markers between TCCs and bicellular contacts (Extended Data Fig. 6a). To quantify spatial asymmetry in the distribution of polarity and luminal markers (ZO-1, aPKC, F-actin, and P-MLC and E-cadherin), fluorescence intensity profiles were extracted from individual cells using Fiji. For each cell, a line vector of 15-px width was drawn from the cell–cell interface to the cell-lumen/fluid pocket interface and the fluorescence intensity values along the line were measured. The distance along each line profile was min–max normalized and rescaled to a relative coordinate ranging from 0 (cell–cell interface) to 1 (cell–lumen/fluid pocket interface) and then discretized into 50 equally sized bins. The mean fluorescence intensity was calculated per bin, and the intensity values were min–max normalized within each profile to focus on the relative spatial distribution rather than the absolute signal intensity. For visualization and analysis, the mean of the raw binned intensities was computed and normalized across embryos for each staining and condition. A polarization index was computed from the normalized binned profiles to quantify relative enrichment at the contact-proximal versus fluid-proximal cell regions (based on ref. ^110^). The polarization index was defined as the ratio between the mean normalized intensity in the fluid-proximal quarter of the profile (outer 25% of bins) and the mean normalized intensity in the contact-proximal quarter (inner 25% of bins) (Extended Data Fig. 7e).

### Statistics and reproducibility

Statistical analyses were performed with GraphPad Prism (v. 10.0) and R (v. 4.2.2) with the packages ggplot2 (v. 3.5.1), ggpubr (v. 0.6.0), broom (v. 1.0.7), car (v. 3.1-3), FSA (v. 0.9.6), multcomp (v. 1.4-28), DescTools (v. 0.99.54), dplyr (v. 1.1.4) and stats (v. 4.2.2). Statistical details are reported in the figure legends. The sample sizes are provided in the figure legends, and no statistical test was used to determine the sample size. The biological replicate is defined as the number of embryos, as stated in the figure legends. No inclusion or exclusion criteria, randomization or blind allocations were applied, and all the analysed samples were included. Unless differently stated in the figure legends, the graphs show mean ± s.e.m., and the error bars are calculated and shown based on the number of cells or embryos, as indicated. The statistical test used to assess the significance is stated in the figure legends and was chosen after testing each group for normality using the Shapiro–Wilk test and for homogeneity of variances using Levene’s test. For comparisons between two groups, a two-tailed Student’s *t*-test was used for parametric distributions with equal variances, a two-tailed Welch’s *t*-test for parametric distributions with unequal variances and a two-tailed Mann–Whitney *U*-test for non-parametric distributions. For multiple pairwise comparisons, analysis of variance followed by Dunnett’s test was used for parametric distributions, whereas a Kruskal–Wallis test followed by Dunn’s test with Dunnett’s adjustment or Bonferroni correction (for pairwise comparisons) was applied for non-parametric distributions. For correlation analysis, Spearman’s correlation was performed. At least three independent experiments were conducted.

### Reporting summary

Further information on research design is available in the Nature Portfolio Reporting Summary linked to this article.

## Online content

Any methods, additional references, Nature Portfolio reporting summaries, source data, extended data, supplementary information, acknowledgements, peer review information; details of author contributions and competing interests; and statements of data and code availability are available at 10.1038/s41567-026-03276-6.

## Supplementary information

Supplementary Information Supplementary Notes I–IV and Figs. 1–8.

Reporting Summary

Supplementary Video 1 Inducing tissue fluidization via optogenetic degradation of α-catenin. Example time-lapse images (2.79-min time intervals) of *Gt(ctnna-citrine)*^*ct3a*^ homozygous embryos during epiboly, without (left) and with (right) opto-zGrad, showing a reduction in α-catenin accumulation and tissue fluidization with no observable off-target effects. Membrane labelled with α-catenin-citrine (green), interstitial fluid with dextran-647 (grey) and nuclei with H2A-BFP (magenta). Scale bar, 50 µm.

Supplementary Video 2 Cell–cell contact dynamics for each material regime. Example time-lapse images (10-s time intervals) from tissues occupying each material regime: solid-like coupled $\alpha$–$\phi$ (wt 50%, pink-shaded rectangle); solid-like uncoupled $\alpha$–$\phi$ (wt 50% + opto-RhoGEF + mannitol, blue-shaded rectangle); fluid-like uncoupled $\alpha$–$\phi$ (wt 50% + Opto-zGrad + ouabain, green-shaded rectangle); and fluid-like coupled $\alpha$–$\phi$ (wt dome + mannitol, orange-shaded rectangle). Membranes labelled with α-catenin-citrine (magenta) and interstitial fluid with dextran-647 (cyan). Scale bar, 50 µm.

Supplementary Video 3 CLFs for each material regime. Example time-lapse images (10-s time intervals) from tissues occupying each material regime; same conditions as those in Supplementary Video 2. The yellow shade indicates individual cell–cell contacts. Membranes labelled with α-catenin-citrine (magenta) and interstitial fluid with dextran-647 (cyan). Scale bar, 20 µm.

Supplementary Video 4 Morphogenesis in coupled versus uncoupled solidification. Example time-lapse images (207.14-s time intervals) of embryos undergoing coupled $\alpha$–$\phi$ solidification (wt margin, left) or uncoupled $\alpha$–$\phi$ solidification (wt margin + mannitol, right) during epiboly stages. Membranes labelled with Lyn-Tomato (green), nuclei with H2B-GFP (magenta) and interstitial fluid with dextran-647 (grey). Scale bar, 50 µm.

Supplementary Video 5 Development of a wt embryo. Representative 3D projection of a light-sheet timelapse (15-min time intervals) of a wt embryo from approximately 30%–60% epiboly (left panel), as well as rotating projections of the start and end time points (middle and right panel, respectively). Cell membrane labelled with α-catenin-citrine (magenta) and interstitial fluid with dextran-647 (green). Scale bars, 100 µm.

Supplementary Video 6 Lumen formation in an explant. Representative 3D projection of a light-sheet timelapse (15-min time intervals) of an explant, with stages corresponding to approximately 30%–60% epiboly (left panel), as well as rotating projections of the start and end time points (middle and right panel, respectively). Cell membrane labelled with α-catenin-citrine (magenta) and interstitial fluid with dextran-647 (green). Scale bars, 100 µm.

Supplementary Video 7 Lumen formation in an opto-RhoGEF + mannitol embryo. Representative 3D projection of a light-sheet timelapse (15-min time intervals) of a wt embryo injected with opto-RhoGEF + mannitol, from approximately 30%–60% epiboly (left panel), as well as the rotating projections of the start and end time points (middle and right panel, respectively). Cell membrane labelled with α-catenin-citrine (magenta) and interstitial fluid with dextran-647 (green). Scale bars, 100 µm.

Supplementary Video 8 Lumen formation in an explant injected with control MO. Representative 3D projection of a light-sheet timelapse (20-min time intervals) of an explant injected with control MO, with stages corresponding to approximately 30%–75% epiboly (left panel), as well as rotating projections of the start and an intermediate (approximately 60% epiboly) time point (middle and right panel, respectively). Cell membrane labelled with α-catenin-citrine (magenta) and interstitial fluid with dextran-647 (green). Scale bars, 100 µm.

Supplementary Video 9 Disrupted lumen formation in an explant injected with Lsr MO. Representative 3D projection of a light-sheet time-lapse image (20-min time intervals) of an explant injected with control MO, with stages corresponding to approximately 30%–75% epiboly (left panel), as well as rotating projections of the start and an intermediate (approximately 60% epiboly) time point (middle and right panel, respectively). Cell membrane labelled with α-catenin-citrine (magenta) and interstitial fluid with dextran-647 (green). Scale bars, 100 µm.

Supplementary Video 10 Disrupted lumen formation in an explant injected with Marveld2a MO. Representative 3D projection of a light-sheet timelapse (20-min time intervals) of an explant injected with Marveld2a MO, with stages corresponding to approximately 30%–75% epiboly (left panel), as well as rotating projections of the start and an intermediate (approximately 60% epiboly) time point (middle and right panel, respectively). Cell membrane labelled with α-catenin-citrine (magenta) and interstitial fluid with dextran-647 (green). Scale bars, 100 µm.

Supplementary Table Description of oligonucleotides.

## Source data

Source Data Fig. 1 Statistical source data.

Source Data Fig. 2 Statistical source data.

Source Data Fig. 3 Statistical source data

Source Data Fig. 4 Statistical source data.

Source Data Fig. 5 Statistical source data.

Source Data Extended Data Fig. 1 Statistical source data.

Source Data Extended Data Fig. 2 Statistical source data.

Source Data Extended Data Fig. 3 Statistical source data.

Source Data Extended Data Fig. 4 Statistical source data.

Source Data Extended Data Fig. 5 Statistical source data.

Source Data Extended Data Fig. 6 Statistical source data.

Source Data Extended Data Fig. 7 Statistical source data.

## Figures and Tables

**Extended Data Table 1:**
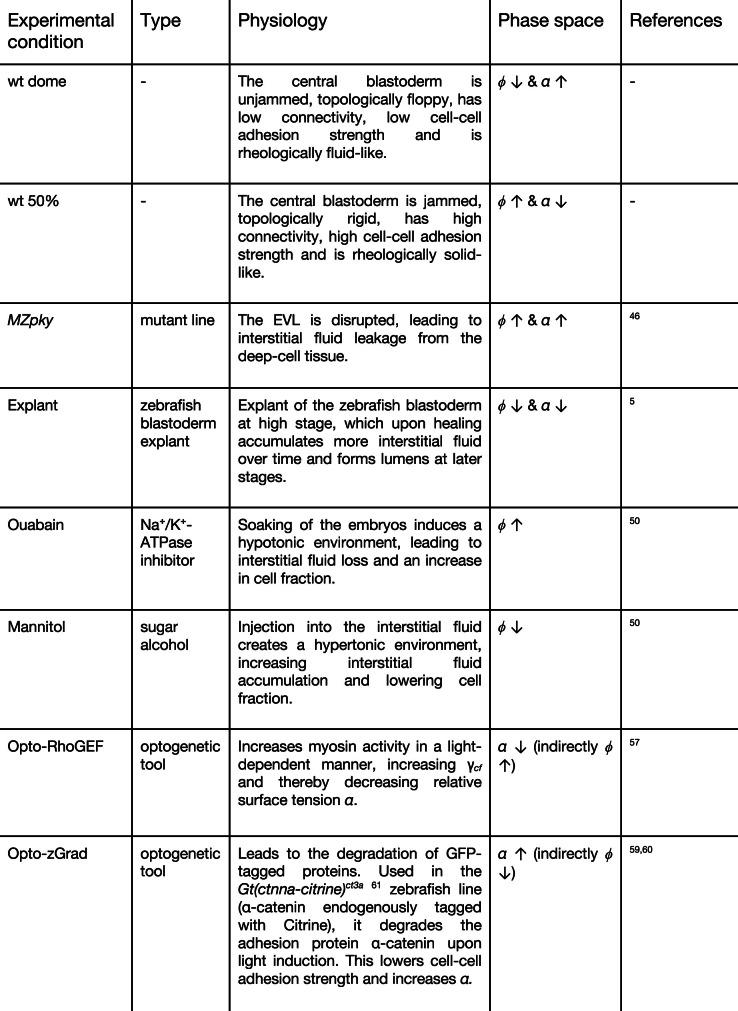
Summary of experimental manipulations

## Data Availability

All statistical source data generated in this study are available in the Supplementary Information. Source data are provided with this paper.

## Code availability

All codes are available via GitHub in the following repositories: rigidity analysis (https://github.com/coldlaugh/pebble-game-algorithm/blob/master/pebble.pyx); optimization of the generated tilings via the Surface Evolver software (https://github.com/bernatze/Rustarazo-Calvo-et-al.-2026-Uncoupling-adhesion--and-density-driven-rigidity...-); Fiji plug-in for connectivity network reconstruction (https://github.com/Petridou-Laboratory/cell-connectivity-networks); and morpho-feature analysis (https://github.com/Petridou-Laboratory/morpho-feature-analysis).
