# Supplementary material for: Adhesion-driven rigidity transition decoupled from density-driven jamming triggers epithelial organization in embryonic tissues
Source: Nat Phys. 2026 Jun 2;22(6):941–53. doi: 10.1038/s41567-026-03276-6 (PMC13271890; doi:10.1038/s41567-026-03276-6)
Supplement: Supplementary file 1 — Supplementary Notes I–IV and Figs. 1–8. [file 41567_2026_3276_MOESM1_ESM.pdf]

nature physics

Supplementary information

https://doi.org/10.1038/s41567-026-03276-6

Adhesion-driven rigidity transition decoupled from density-driven jamming triggers epithelial organization in embryonic tissues

In the format provided by the authors and unedited

Table of contents

Supplementary Note……2
I. Cell-cell contact energy……2
II. Adhesion-induced rigidification……3
A. Generic rigidity……3
B. Generic rigidity arising from cell-cell adhesion changes……4
III. Adhesion-induced collapse of porosity……7
A. Porosity……7
B. Maximal cell fractions……10
IV. Numerical Solutions……10
A. Random seeds for arbitrary cell tilings……11
B. Adhesion-induced rigidification……14
C. Adhesion-induced confluency……15
D. Computing the rigid clusters……16

I. CELL-CELL CONTACT ENERGY

We study non-confluent tissues, where cells are in contact among them and with the interstitial fluid. We are interested in cell-fluid interface and cell-cell contacts. To that end, we assume that the energy of the system can be approximated by the Hamiltonian of a soap bubble [1, 2]:

$$\mathcal{H} = \gamma_{cc} \sum_{i<j} w_{ij} + \gamma_{cf} \sum_{i} a_i + \tilde{K} \sum_{i} (V_i - V_0)^2 \quad , \tag{1}$$

being the first sum performed over all cell-cell potential contacts, w_{ij} the contact area between cells i and j, a_i the area of cell i exposed to the interstitial fluid, $\tilde{K}$ the compressivity constant, V_i the volume of the cell and V_0 the volume of the cell at rest. We assume V_0 to be constant and $\tilde{K} \gg \gamma_{cc}, \gamma_{cf}$, such that the cells are considered incompressible and thus the volume/area can be imposed as a boundary condition. Throughout the paper we consider that cell contacts are governed by Young-Dupré's law. This law states that, in a force-balance situation, the contact angle between the membranes of two cells in contact, θ, and the relation between the strength of the cell-cell surface tension, γ_{cc}, and cell-fluid surface tension, γ_{cf}, are related through [2–4]:

$$\frac{\gamma_{cc}}{\gamma_{cf}} = 2\cos\left(\frac{\theta}{2}\right) \quad .$$

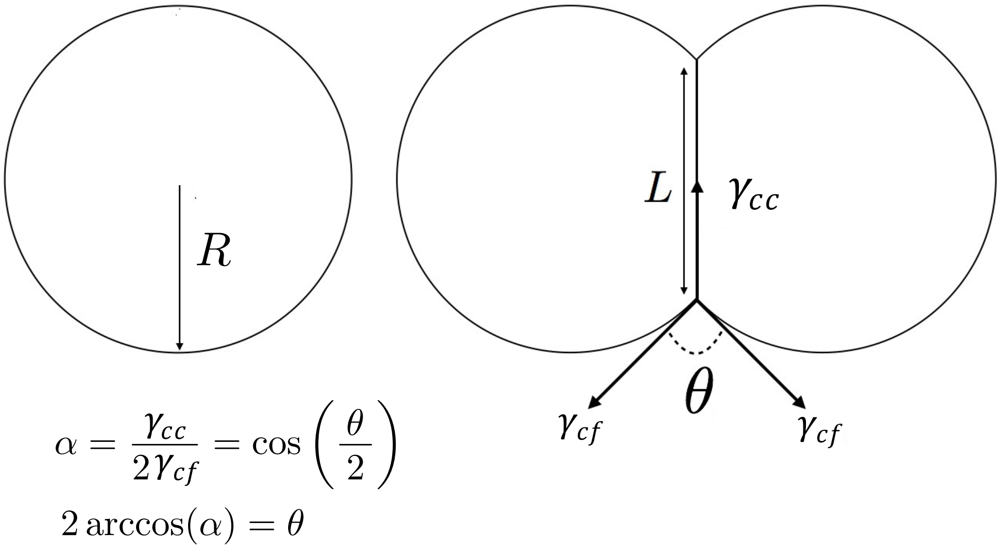

Suppl. Note Fig. 1: Schematic representation of the Young-Dupré relation.

In Suppl. Note Fig. (1) we detail schematically each of these terms. From the above

relation, one can derive a non-dimensional parameter, α, describing the relative strength of the cell-cell surface tension, γ_{cc}, and cell-fluid surface tension, γ_{cf}:

$$\alpha = \frac{\gamma_{cc}}{2\gamma_{cf}} = \cos\left(\frac{\theta}{2}\right) \quad , \tag{2}$$

and, consequently, by defining $K = \tilde{K}/2\gamma_{cf}$, re-write the Hamiltonian in a non-dimensional form:

$$\mathcal{H}(\alpha) = \alpha \sum_{i<j} w_{ij} + \frac{1}{2}\sum_i a_i + K \sum_i (V_i - V_0)^2 \quad . \tag{3}$$

If we consider conservation of area/volume as a boundary condition, we are effectively led to:

$$\mathcal{H}(\alpha) = \alpha \sum_{i<j} w_{ij} + \frac{1}{2}\sum_i a_i \quad . \tag{4}$$

Key to all this framework is that the non-dimensional parameter α can be inferred from the cell-cell contact angles, an observable that can be empirically extracted. This enables us to perform predictions on structural properties of the arrangements of cells in contact only considering geometric grounds. Such derivations are valid in a context with no net stresses applied over the tissue.

II. ADHESION-INDUCED RIGIDIFICATION

A. Generic rigidity

We will use the concept of *generic rigidity*, a graph-theoretic concept [5]. Suppose a network $G'(V', E')$ made of a set V' of nodes and a set E' of links between nodes. A *spanning subgraph* is a subgraph $G(V, E)$ of $G'(V', E')$ by which $V = V'$ and $E \subseteq E'$. An *induced subgraph* $g(V_g, E_g)$ of $G'(V', E')$ is a subgraph by which $V_g \subseteq V$ and in which all the links between elements of V_g existing in G' are present. Finally, a graph is *connected* if there is a path of finite length between any pair of nodes of V'.

As we work using 2D projections, we ground our reasoning on the plane. Consider that links impose spatial constraints. In d dimensions, two nodes $v_i, v_j \in E$ will have a priori d spatial degrees of freedom each, but if $\{v_i, v_j\} \in E$, that is, there is a link between v_i and v_j, then the degrees of freedom of v_i and v_j will be at most $d-1$, because the link acts as spatial constraint for the two nodes. A network is called generically rigid if the set of

links absorbs all the degrees of freedom of the nodes in d dimensions, such that nodes do not have independent movements. This implies that, if nodes are interpreted as rigid bars or springs at rest, no deformations of the structure are possible at no energy cost; i.e., the Young modulus of the whole network is > 0 [6]. In general, a network $G'(V', E')$ embedded in a 2-dimensional space will be generically rigid if there is a connected, spanning subgraph $G(V, E)$, with $E \subseteq E'$, by which:

1. $|E| = 2|V| - 3$

2. For every subset $V_g \in V$, with $|V_g| \geq 2$, the induced subgraph g (with link set $E_g \subseteq E$) is such that $|E_g| \leq 2|V_g| - 3$.

This theorem is due to Geiringer [7] and Laman [8]. We emphasize that generic rigidity is a purely topological property of the network.

With these tools in hand, we will look at a specific graph, a rhombus –see Suppl. Note Fig. (2A). In this case, it is easy to check that the rhombus is not generically rigid because even if we consider the whole graph, then: $|E'| = 4$, $|V'| = 4$, and, thus, $|E'| < 2|V'| - 3$, forbidding the existence of a spanning subgraph satisfying the first condition of Geiringer-Laman's theorem. In the case of the graph of Suppl. Note Fig. (2B,C), with an accessory link connecting the central nodes, $|E'| = 5$, and the whole graph itself satisfies the first condition of Geiringer-Laman's theorem. A simple counting argument throughout all the potential configurations can be used to check that any induced subgraph satisfies the second condition of Geiringer-Laman's theorem. Therefore, the graph in A/ is floppy and the ones in B/ and C/ are generically rigid.

B. Generic rigidity arising from cell-cell adhesion changes

We want to explore how the reduction in α may affect the topology of cell-cell contacts and, in consequence, the potential rigidity properties. To that end, we use the toy motif of 4 cells in a 2D projection that is analytically tractable. This will allow us to determine a critical point in α. Further, using numerical simulations, we will see that the result scales to arbitrary cell arrangements.

Let us suppose that we have a floppy motif made of 4 cells –see Suppl. Note Fig. (2D). The network of cell contacts corresponds to the one shown in see Suppl. Note Fig. (2A),

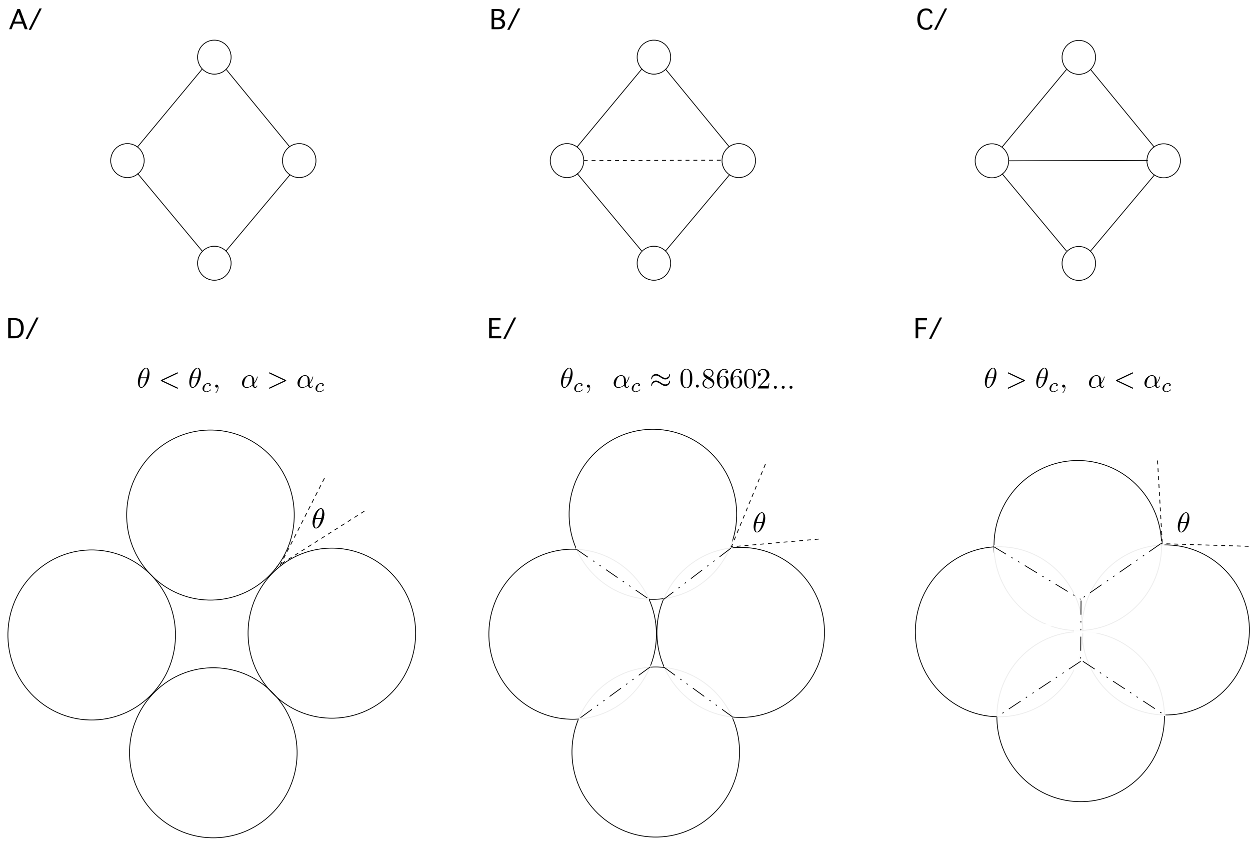

Suppl. Note Fig. 2: Topological and geometric effects of the decrease of α.

for which we have shown it is not generically rigid. We want to know at what α the motif rigidifies. That is: at which α a new contact is created such that its cell-cell contact network corresponds to the one shown in Suppl. Note Fig. (2B,C).

To that end, we start with a symmetric, rhombus-like configuration of 4 disk shaped cells with only pointwise contacts –see Suppl. Note Fig. (2A,D)– of radius 1 located at $(-\sqrt{2}, 0), (\sqrt{2}, 0), (0, -\sqrt{2}), (0, \sqrt{2})$, respectively. To mimic the effect of the increase of adhesion, we will progressively bring the disks located left and right of the vertical axis closer and closer, allowing overlap. The new configuration of the centers of the cells will be $(-\sqrt{2} + \delta, 0), (\sqrt{2} - \delta, 0), (0, -\sqrt{2}), (0, \sqrt{2})$, respectively, and we keep the radius invariant. The bisectrix line between two overlapping circles will represent the cell-cell contact region without any loss of generality, as we are only interested in the angle between the two circles and, therefore, conservation of area is not required, although it can be achieved by just applying a rescaling, which would not affect the configurations of angles. The disks on top and at the bottom of the rhombus remain fixed. Eventually a new contact is created, when

the location of the centers of mass is $(-1,0),(1,0),(0,-\sqrt{2}),(0,\sqrt{2})$, leading to $|E| = 5$ and, therefore, according to the derivation provided in the previous section, (generically) rigidifying the motif –see Suppl. Note Fig. (2B,E,F). Now it remains to compute the angle between circles that results from the obtained configuration. From that, we can straightforwardly, applying Young-Dupré's law, compute α_c. The critical point at which the new contact is formed corresponds to:

$$\alpha_c \approx 0.86602... \quad . \tag{5}$$

The whole computation is based on finding the θ_c using standard geometric considerations. In Suppl. Note Fig.(3) we detail the reasoning.

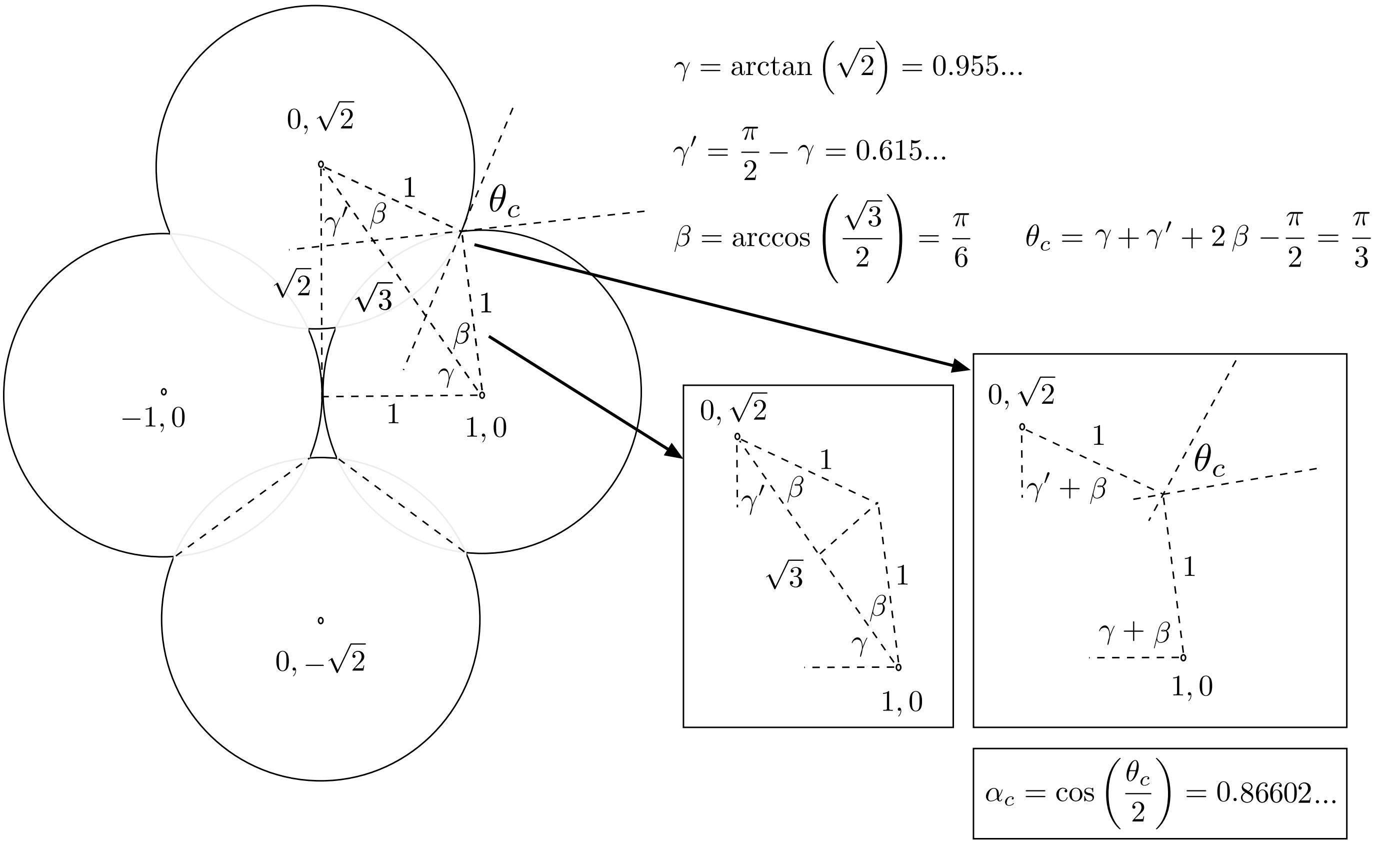

Suppl. Note Fig. 3: Computing the angle and the α triggering the internal contact and, therefore, rigidity.

III. ADHESION-INDUCED COLLAPSE OF POROSITY

In this section we will show how the rigidification for values $\alpha < \alpha_c$ explained above also entails the emergence/collapse of porosity, that is, that all the interstitial voids become closed in the minimum energy state.

A. Porosity

To study the effect of the reduction of the α on the porosity of the tissue, we follow a strategy based again on the fact that, in the 2D projection, cells in contact can be treated as two disks whose contact region is the bisection of intersection between both. We consider a starting point where two disks of radius R are separated by a distance $2R$, having, therefore, only one contact point –see Suppl. Note Fig. (4). One can study the evolution of α by applying the following rescaling:

$$R \to \lambda R\,; \quad \lambda \geq 1 \quad ,$$

while keeping the distance of the centers of mass invariant. We refer to λ as the *scaling parameter*. In that context, the actual units become rescaled. However, there is no need to unfold the scaling, as long as we are interested in i) Cell fraction and ii) Intersection angles to infer the critical α derived from the Young-Dupré relation. Metaphorically speaking, we are inflating the cells while keeping the centers of mass static. Considering an arbitrary radius, for $\lambda \geq 1$, we have that the intersection angle between two adjacent cells can be inferred as:

$$\theta = 2\arccos\left(\frac{1}{\lambda}\right) \quad ,$$

from which we conclude that:

$$\alpha = \frac{1}{\lambda} \quad . \tag{6}$$

In Suppl. Note Fig. (4) we detail this observation.

The key point in the computations is to find at which point a 3-cell junction is formed –see Suppl. Note Fig. (5). Indeed, when 3-cell junctions are favored, cells will tend to pack in confluent regions. In particular, using the scaling parameter λ, one can see that:

- For $\lambda < 2/\sqrt{3}$, 3 cells in contact forming a triangle do not create spontaneously a 3-cell junction

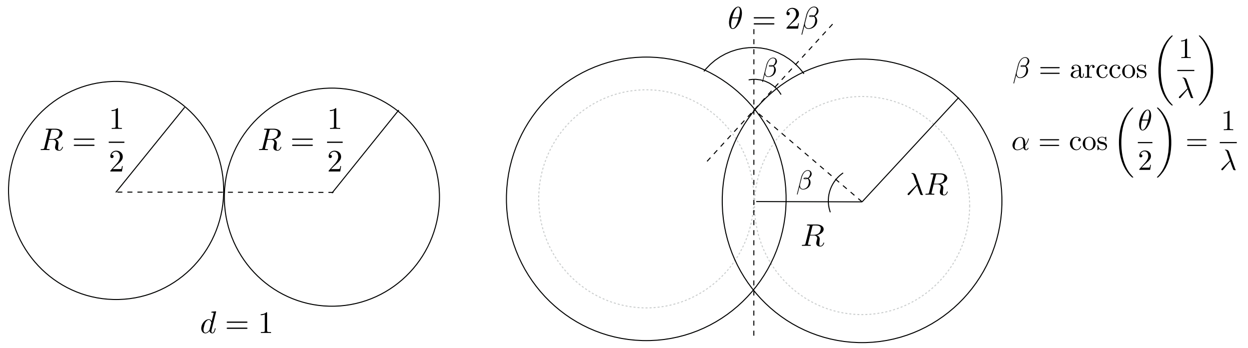

Suppl. Note Fig. 4: Schema detailing the use of the scaling parameter λ.

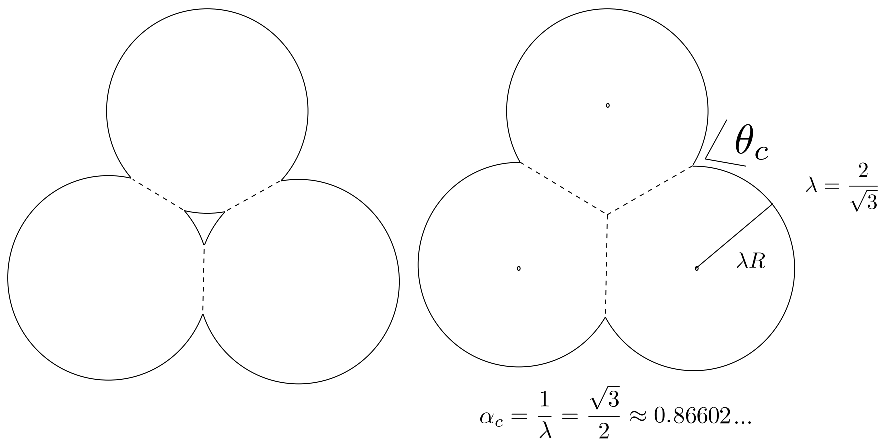

Suppl. Note Fig. 5: Collapse of the interstitial network: Computing the critical α for closing a 3-cell junction hole using the rescaling strategy described in the theorem.

- $\lambda \geq 2/\sqrt{3}$, 3 cells in contact forming a triangle create spontaneously a 3-cell junction

To see that, we reason as follows: As long as the radius of the cell is larger than the distance from any of the cells to the centroid of the triangle, the gap is closed. In consequence, to compute the critical λ beyond which the hole is closed, the only thing we need to know is the distance between any cell and the centroid of the triangle. Considering that the 3-cell arrangement is symmetrically distributed, this critical radius (R_c) turns out to be:

$$R_c = \frac{1}{\sqrt{3}} \quad ,$$

which implies $\lambda = \frac{2}{\sqrt{3}}$. In Suppl. Note Fig. (5) we show the reasoning schematically. Thanks to equation (6) we can map this rescaled radius into the physically significant parameter α, leading to a critical point for confluency α_c of:

$$\alpha_c = \frac{\sqrt{3}}{2} \approx 0.86602... \quad (7)$$

which, interestingly, coincides with the critical point of rigidity. This implies that *the emergence of adhesion-induced rigidity, as computed in section II B of this suppl. note, occurs at the same critical point of adhesion strength as the emergence of the adhesion-induced confluency/collapse of porosity.* Simulations using large-scale disordered arrangements of cells simulating tissues show a behavior consistent with these predictions. Equivalent results for the threshold for confluency predicted in eq. (7) were previously reported in [9, 10]. We here extended the scope of the result using numerical simulations with arbitrary cell tilings and showing that the phenomenon of pore closing –and, in consequence, the transition to confluency– is expected in generic cell arrays. In addition, the derivation strategy allows us to compute the maximum cell fractions for all values of α, which is the target of the next subsection.

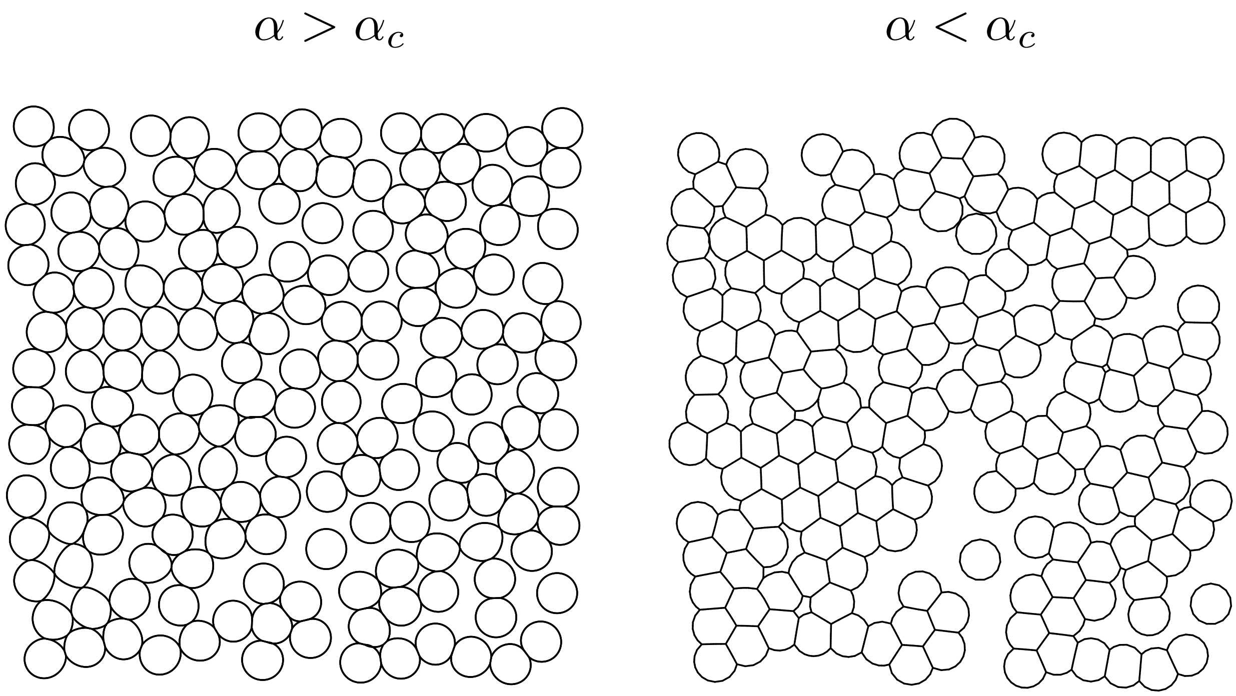

Suppl. Note Fig. 6: Simulations of real tissues at $\alpha > \alpha_c$ and $\alpha < \alpha_c$. We observe that, below α_c (right), almost all 3-cell junctions are closed. We used the same cell underlying arrangement for both simulations. α's used: $\alpha = 0.93$ (left), $\alpha = 0.83$ (right).

B. Maximal cell fractions

Start with a hexagonal tiling of hard disks of equal radius, as it is known that this is the arrangement leading to the maximal disk density. We then apply the following strategy: For each triangular junction, we consider the surface of triangle formed by the three centers of mass of three cells and how this is getting filled along the increase of λ. Considering the fact that the triangle contains 1/6 of each of the 3 cells and the 1/2 of the intersection surface shared by each pair of cells, we have that the relative surface, ϕ, occupied by the cells within the triangle is:

$$\phi = \frac{1}{\mathrm{A}(T)}\left(\frac{1}{2}\int_S dS - \frac{3}{2}\int_\cap dS\right) \quad ,$$

We assume that the circles for $\alpha = 1$ have radius $R = 1$. This leads to an equilateral triangle of side 2, being its overall surface of the $\mathrm{A}(T) = \sqrt{3}$. After applying a rescaling operation $R \to \lambda R$, the other terms read:

$$\int_S dS = \pi\lambda^2 \; ; \qquad \int_\cap dS = 2\left(\lambda^2 \arccos\left(\frac{1}{\lambda}\right) - \sqrt{\lambda^2 - 1}\right) \quad .$$

In terms of α, using that $\alpha = \frac{1}{\lambda}$:

$$\phi(\alpha) = \frac{\sqrt{3}}{\alpha^2}\left(\frac{\pi}{6} - \arccos(\alpha) + \alpha\sqrt{1-\alpha^2}\right) \; , \quad (\alpha \geq \alpha_c) \quad . \tag{8}$$

It is easy to check that $\phi(1) \approx 0.907$, as it is well known for the maximum density of the packing of hard disks of equal radius; and that $\phi(\alpha_c) = 1$. The above equation tells us that full confluency is only possible, for tissues under force balance conditions, at values $\alpha \leq \alpha_c$. In Suppl. Note Fig. (7) the evolution of this maximum density is shown.

IV. NUMERICAL SIMULATIONS

Numerical simulations have been performed using the C based software `Surface Evolver` version 2.70 [11]. To study the evolution of a 2D cell tiling using `Surface Evolver`, it is necessary to enter an initial *mosaic* –eventually with non-covered regions– specifying the location of the cells, a primary polygon-like geometry and the initial topology of cell-cell contacts. Later this initial condition will evolve 1/ By increasing the resolution of the perimeter of the cells –thereby achieving realistic geometries– and 2/ Optimizing the global

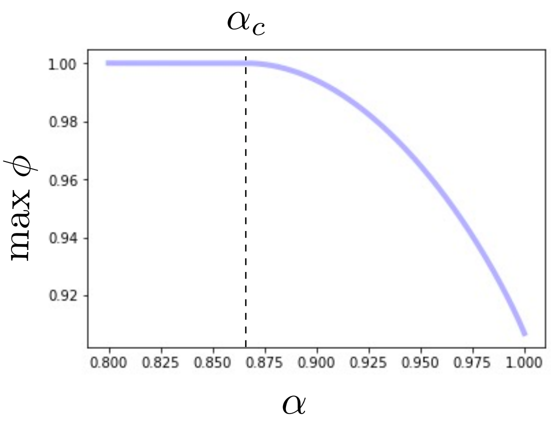

Suppl. Note Fig. 7: Maximum achievable cell fraction ϕ as a function of α according to eq. (8).

geometry of individual cells and size of cell-cell contacts according to a global soap-bubble-like Hamiltonian like the one described in equation (3), in a relaxation process towards the desired α parameter.

Throughout this research we do not consider the action of external pulling or pushing forces and, thereby, we expect the tilings to be in equilibrium with respect to the soap-bubble Hamiltonian.

A. Random seeds for arbitrary cell tilings

The definition of the initial random seed that will be further optimized to the desired α can be performed following different algorithmic procedures: 1/ The Lubachevsky-Stillinger algorithm, indicated to study jamming of hard disks 2/ Random location of disks in a plane, indicated to generate floppy –subcritical– cell arrangements with already existing contacts and 3/ a network-like based algorithm, in which the network topology is a-priori given, indicated for fine-grained exploration towards confluency. The density of these initial seeds is initially parametrized through an estimation. Since the optimization process may induce variations, the cell density is re-computed at the end of the process.

1. Lubachevsky-Stillinger algorithm (LSA) –Jamming

The Lubachevsky-Stillinger (compression) algorithm (LSA) is the standard numerical method used to simulate the physical process of compressing an assembly of hard disks that

eventually leads to hard-sphere-like jamming [12]. In this research we used the C++ based implementation provided in [13]. Although the LSA method allows us to generate random tilings at different densities, most of the contacts between cells are formed at the limit of the jamming transition $\phi_c \approx 0.84$. In consequence, the LSA may not be indicated for producing the seeds for random tilings at subcritical densities.

2. *Random disk spreading (RDS) –Topological consequences of changes in* α

The method described below allows us to generate random tilings with subcritical target densities ϕ_T, and is based on the RSA algorithm reported in [14] to generate subcritical random disk arrays. This method is indicated for the study of rigidification of previously floppy structures upon decrease of α. The steps followed in the algorithm are described below:

- Over an a-priori defined square of length L –in units of cell diameter $D = 2R$, where R is the average cell radius– send N' random possible coordinates $\vec{x_1}, ..., \vec{x_{N'}}$.
- The sequential process of generation of random coordinates is subject to a selection criterion: If when generating the k-th random coordinate an already existing coordinate $\vec{x_i}$, $i < k$ is such that $d(\vec{x_i}, \vec{x_k}) < (1 - \epsilon)2R$ this coordinate $\vec{x_k}$ is discarded, as it would lead to a large overlapping pair of disks.
- If the number of accepted generated coordinates N ($N \leq N'$) reaches a value such that, $\phi_T \leq N\phi R^2/L^2$, where ϕ_T is the target density, the process stops, since the target density has been achieved.
- If after a long number of iterations the target density cannot be achieved, we perform a random search along the area identifying possible empty spaces that can be filled using the previous distance conditions. Note that for densities close to ϕ_c this process is harder and harder and becomes technically unfeasible for $\phi \approx \phi_c$, where the LSA or the network based algorithm for generating mono size tilings defined below are more indicated.
- Finally build a collection $C_1, ...C_N$ of disks centered on each of the accepted coordinates $(\vec{x_1}, ..., \vec{x_N})$. For any pair of disks C_i, C_k such that $d(\vec{x_i}, \vec{x_k}) \leq 2R$ we define a contact.

Since the presence of overlaps and further changes on adhesion may slightly alter the cell fraction, we may encounter the situation by which the actual achieved density $\phi < \phi_T$. If this happens, we need to generate more disks, refining the halting condition and rewriting it as $\phi \leq (N + \delta)\phi R^2/L^2$ to achieve the desired densities $\phi \approx \phi_T$ after optimization.

In the case that the target density is not achieved through the proposed RDS algorithm, we run an advanced version of it, introducing the possibility of randomly introducing smaller disks. Specifically, after the initial run of the RDS algorithm, the domain is systematically scanned to identify unoccupied regions that were not detected during the random search. When possible empty spaces are identified which may be too small for the distance condition, a new distance condition can be introduced: $\rho R < d(\vec{x_i}, \vec{x_k}) < (1 - \epsilon)2R$. This procedure results in a slightly polydisperse tiling, since a small fraction of the disks may be smaller than the initial size to fit existing gaps. In our simulations, we used $\rho = 1.3$, and new disks were inserted with a reduced radius $R^* = 0.85R$ for larger gaps or $R^* = 0.6R$ for smaller gaps. Panels Fig. 1k and Fig. 1o of the main text were generated using this advanced version of the RDS algorithm.

3. Network-based tilling generation (NTG) —transition to confluency

The following method is indicated for the generation of dense tilings and/or when we want to study the transition from non-confluent to confluent tissues upon decrease of α. The steps are the following:

- Generate a regular triangular lattice with a given size –side L cells with radius R. The position of the N nodes is $(\vec{x_1}, ..., \vec{x_N})$. In this setting, adjacent nodes are at exactly distance $2R$.
- Remove some sites with probability p, computed from the desired target density ϕ_T.
- Introduce noise in the geolocalization of the nodes. Position $\vec{x_k} = (v_k, u_k)$ is replaced by $\vec{x_k}^\zeta = (v_k + \zeta, u_k + \zeta')$ where ζ, ζ' are random numbers drawn from a Gaussian distribution centered at 0 whose standard deviation is $\sigma = \epsilon R$.
- For each $(\vec{x_1}^\zeta, ..., \vec{x_N}^\zeta)$ draw a circle of radius R around it.
- For each pair $\vec{x_i}^\zeta, \vec{x_k}^\zeta$, establish a contact if $d(\vec{x_i}^\zeta, \vec{x_k}^\zeta) < 2R + \delta$.

B. Adhesion-induced rigidification

We detail here how initially floppy tilings at subcritical target density ϕ_T can become generically rigid by decreasing the parameter α using the `Surface Evolver` software (version 2.70) [11]. Since we are working with subcritical densities and we want to observe changes in the topology, we use the above described RDS method to generate the seed which will define the starting point of the optimization process.

The parameter t sets the minimum segment length forming a polygon –which represents a cell–, thus controlling also the number of segments a cell is made of. As in previous sections, we consider 2D projections of cell arrangements, where each cell is described by a set of vertices. The method followed in this case is:

- Generate a random, subcritical tiling with target density $\phi_T < \phi_c$ and the desired size with the RDS method.
- Stabilize the tiling in the hard-sphere regime ($\alpha = 1$).
- Decrease α in a quasi-static way until the desired α is reached. In this step, the mechanism of re-meshing implemented by the `Surface Evolver` acts by alternatively reducing and increasing the resolution of the geometry of the cells. This is done by merging all segments lower than a certain length (t parameter) and subsequently dividing such segments by 2. With this, the optimization algorithm can explore the potential configurations minimizing the global surface energy in an efficient way. According to our simulations, working with very fine grained geometries (low t parameters) all the time may trap the system in local optima. In consequence, we need to introduce sporadically large merging events (large t's) to trigger topological changes. To preserve the high resolution of the simulation but, at the same time, allow topological rearrangements, we apply the re-meshing t-parameter as following a Weibull function:

$$p(t) = \frac{k}{\lambda}\left(\frac{t}{\lambda}\right)^{k-1} e^{-(t/\lambda)^k} \quad .$$

This allows us to perform most of the re-meshing events around a well-defined mean, keeping high resolution, but, from time to time, massive re-meshing events allow topological rearrangements.

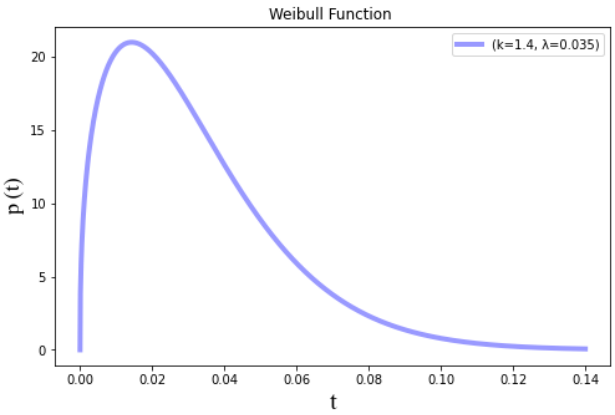

Suppl. Note Fig. 8: Weibull function for k=1.4 and λ=0.035

- When the targeted α is reached and no changes in the energy are appreciated throughout successive rounds of optimization, we end the process. The result is a tiling with the desired α and approximately the target density ϕ_T. Since the density can be altered, it is recomputed after the optimization process.

C. Adhesion-induced confluency

To test the mathematical prediction on the emergence of adhesion-induced confluency in tissues at $\alpha_c \approx 0.86602$ (see the mathematical section), we perform several simulations considering different cell arrangements. As a starting point, we study one of the simplest topologies: a triangular motif of 3 symmetrically distributed cells of equal radius in contact. The simulation starts with cells modeled as non-adhesive disks at $\alpha = 1$, which is progressively decreased to $\alpha = 0.7$. Using the same initial configuration, we test different values of the resolution parameter t. Since the computation of the angle formed requires high resolution –to be as close as possible to a circle– we need to use very low t values. For these values of t we observe that at α_c the interstitial hole between cells is closed, leading to the formation of a tricellular contact (TCC) in which the three cells involved share a vertex.

To extend the analysis to tissues, we generate both ordered and disordered arrays of cells (of approximately 10x10 cells in size). We use the NTG method, described in the previous section, to construct the seed for the simulations. Simulations of the evolution of these tilings upon reduction of α are performed for different t analogously to the simple triangular

case (from $\alpha = 1$ up to $\alpha = 0.7$). The percentage of TCCs in a cell tiling is then:

$$\text{TCC} = \frac{N_{\text{TCC}}}{N_{\Delta}}, \tag{9}$$

where N_{TCC} is the number of closed interstitial holes between 3 cells and N_{Δ} is the number of triangles in the contact network, respectively, which can be computed directly from the adjacency matrix of the network A, defined as:

$$A_{ij} = \begin{cases} 1 \text{ if node } i \text{ and } j \text{ connected} \\ 0 \text{ otherwise} \quad , \end{cases}$$

being the amount of triangles computed as [15]:

$$N_{\Delta} = \frac{1}{6}\text{tr}A^3 \quad .$$

In both arrangements, for $t = 0.01$ a sharp transition occurs in the percentage of TCCs in the proximity of α_c, indicating that below α_c the majority of holes are closed, in agreement with the mathematical predictions. For $t < 0.01$ this transition is not observed, possibly due to the existence of local minima trapping the system due to the small size of the segments.

D. Computing the rigid clusters

Identification of floppy and rigid areas of the cell-cell contact networks was performed using `pebble.py` [16], available at:
https://github.com/coldlaugh/pebble-game-algorithm/blob/master/pebble.pyx

[1] T. Hayashi and R. W. Carthew, Nature **431**, 647 (2004).

[2] D. L. Weaire and S. Hutzler, *The Physics of Foams* (Clarendon Press, 1999).

[3] J.-L. Maître, H. Berthoumieux, S. F. G. Krens, G. Salbreux, F. Julicher, E. Paluch, and C.-P. Heisenberg, Science **338**, 253 (2012).

[4] J.-L. Maître, R. Niwayama, H. Turlier, F. Nédélec, and T. Hiiragi, Nature cell biology **17**, 849 (2015).

[5] D. J. Jacobs and M. F. Thorpe, Phys. Rev. Lett. **75**, 4051 (1995).

[6] M. van Hecke, Journal of Physics: Condensed Matter **22**, 033101 (2009).

[7] H. Pollaczek-Geiringer, ZAMM-Journal of Applied Mathematics and Mechanics/Zeitschrift für Angewandte Mathematik und Mechanik **7**, 58 (1927).

[8] G. Laman, J Eng Math **4**, 331 (1970).

[9] S. E. Parent, D. Barua, and R. Winklbauer, Biophysical Journal **113**, 913 (2017).

[10] S. Kim, M. Pochitaloff, G. Stooke-Vaughan, and O. Campás, Nature Physics **17**, 1 (2021).

[11] K. Brakke, Experimental Mathematics **1**, 141 (1992).

[12] B. Lubachevsky and F. Stillinger, J Stat Phys **60**, 561â583 (1990).

[13] M. Skoge, A. Donev, F. H. Stillinger, and S. Torquato, Phys. Rev. E **74**, 041127 (2006).

[14] E. L. Hinrichsen, J. Feder, and T. Jøssang, Phys. Rev. A **41**, 4199 (1990).

[15] M. Newman, *Networks* (OUP Oxford, 2018).

[16] L. Zhang, D. Z. Rocklin, B. G.-g. Chen, and X. Mao, Phys. Rev. E **91**, 032124 (2015).
